# Later life learning: what is it, and who is it for? A systematic scoping review of the “learning and health in later life” literature

**DOI:** 10.1093/geront/gnaf283

**Published:** 2025-11-24

**Authors:** Ourania Sfakianaki, Nick Shryane, Antony Payton, Laura J E Brown

**Affiliations:** Social Statistics Department, School of Social Sciences, The University of Manchester, Manchester, United Kingdom; Social Statistics Department, School of Social Sciences, The University of Manchester, Manchester, United Kingdom; Division of Informatics, Imaging and Data Sciences, School of Health Sciences, The University of Manchester, Manchester, United Kingdom; Manchester Centre for Health Psychology, Division of Psychology and Mental Health, School of Health Sciences, The University of Manchester, Manchester Academic Health Sciences Centre, Manchester, United Kingdom

**Keywords:** Learning dimensions, Measurement of learning, Sociodemographic disparities

## Abstract

**Background and Objectives:**

There is growing evidence for the health benefits of later life learning (LLL). However, there is considerable variety in how LLL has been operationalized and/or measured across studies, making it difficult to determine what the active ingredients of LLL are. There may also be underrepresented groups of participants in this research. This scoping review aimed to map existing research on LLL and health in order to develop a classification framework for reporting LLL studies, and identify the demographic characteristics of participants included in this research.

**Research Design and Methods:**

The researchers systematically searched CINAHL, MedLine, PsycINFO, ERIC, Open Access Theses and Dissertations, and OpenGrey for empirical studies on LLL’s effects on health outcomes.

**Results:**

A total of 51 articles met the inclusion criteria. The extracted data were used to describe the studies according to a classification framework comprising eight core LLL dimensions: organizer, target audience, format, content, instruction method, age, duration, and frequency. Notable gaps were identified, including the underrepresentation of men and insufficient reporting on participants’ education, marital status, socioeconomic status, and ethnicity.

**Discussion and Implications:**

The findings highlight inconsistencies in LLL’s operationalization and/or measurement, alongside gaps in sociodemographic data reporting, that make it difficult to draw generalizable conclusions about the effects of LLL on health. The classification framework the researchers developed provides a tool to describe and synthesize findings across studies to better understand the mechanisms underlying LLL and health. Future research should also explore LLL’s effects on older male learners who have been underrepresented in past research.

## Background

As populations age worldwide, identifying modifiable factors that promote healthy aging has become a key research priority. There is increasing evidence that later life learning (LLL) is one such factor that is associated with improved health outcomes. For example, participation in learning activities has been associated with significant reductions in anxiety and depression among older adult learners (Bužgová et al., 2024). LLL has also been found to have a positive impact on cognitive health-related outcomes, such as better global cognition and brain functional connectivity (Bubbico et al., 2019). Studies have further demonstrated important physical health-related benefits of LLL, including improvements in arterial stiffness (Uemura et al., 2021) and respiratory muscle strength (Fu et al., 2018). Engagement in LLL has also been associated with improved well-being, as evidenced by enhanced self-esteem and increased social engagement (Santini et al., 2020), both of which are critical determinants of healthy aging (World Health Organization [WHO], 2015).

However, there are some key issues in the literature that make it difficult to determine the specific mechanisms that underlie these apparent effects of LLL on health. First, there is no consistent way in which LLL has been described and defined. For instance, variations in provisions for LLL over time have led researchers to use a range of terms, such as “lifelong learning,” “continuing education,” and “third age education,” to describe LLL and its core principles (Findsen & Formosa, 2016). More importantly, the particular activities that are considered to represent LLL vary widely, ranging from university courses (Lenehan et al., 2016) and self-directed study (Sloane-Seale & Kops, 2010), to memberships in sports clubs, gym and exercise classes (Jenkins & Mostafa, 2015) and game-based learning programs (Hsu et al., 2023). These activities differ on important dimensions, such as the cognitive load of the activity involved (Illeris, 2002) and the presence of other factors that have known effects on health, such as physical and social activity (Bielak & Gow, 2023). Furthermore, the age criteria for older learners vary significantly, with some studies including participants aged 50 years and above (Hardy et al., 2017), while others focus specifically on individuals over 65 years (MacRitchie et al., 2019). Learning activities also differ in frequency and duration, with some studies examining persistent participation in LLL (Jenkins & Mostafa, 2015) and others focusing on periodic engagement, such as 12-week learning programs (Valis et al., 2019). This variability makes it difficult to determine which (if any) components of LLL are responsible for the health and well-being effects that are observed.

Another key issue in the literature relates to the demographic groups that have been included. Studies on LLL often reveal sociodemographic biases, with certain groups, particularly older adults from lower socioeconomic backgrounds or non-White ethnic backgrounds, being underrepresented. For example, a study of the Osher Lifelong Learning Institutes in the United States revealed that participants were primarily White, female, married, and highly educated (Wenzel et al., 2024). Gender differences are also evident in the types of activities undertaken, with women often participating more in nonformal and social learning activities, while men are more likely to engage in formal education (Jenkins & Mostafa, 2015). Identifying and addressing sociodemographic disparities is crucial, as they can limit the generalizability of findings and obscure the potential benefits of LLL for underrepresented groups of older adults.

### Objectives

The aim of this scoping review was to map and synthesize research that has focused on the relationship between learning and health in later life, in order to answer the following questions: (a) What terms have been used to describe LLL in research on the effects of LLL on health outcomes? (b) How has LLL been operationalized and/or measured in these studies, and what were the health-related outcomes that have been investigated? Specifically, which learning activities were included; at what age(s) did these learning activities take place; and what was the duration and frequency of these learning activities? (c) What are the sociodemographic characteristics of the older learners who participated in these studies? These data were used to develop a classification framework that can be used to describe key parameters of LLL studies, and to identify specific underrepresented groups of older adults in LLL research.

### Research design and methods

A scoping review methodology was chosen for its suitability in addressing broad research questions and mapping heterogeneous literature (Levac et al., 2010). It is particularly effective for clarifying key components, identifying research gaps, and guiding future research (Munn et al., 2018). The review process followed the preferred reporting items for systematic review and meta-analysis extension for scoping reviews (PRISMA-ScR; Tricco et al., 2018). A protocol (Sfakianaki et al., 2022) was developed and preregistered on the Open Science Framework (https://doi.org/10.17605/OSF.IO/FGVW3).

### Eligibility criteria

Eligible studies for inclusion were empirical (observational or interventional) studies published in English that aimed to explore the relationship between LLL and health. Specific inclusion criteria were defined using the Patient problem or Population, Intervention, Comparison or Control, Outcome (PICO) approach.

### Population

Studies had to include an adult population aged over 50 years. While many studies set higher limits of 60 or 65 years as the start of “later life,” lower age limits of 50–55 years are sometimes used in studies of later life in developing countries (Findsen & Formosa, 2016). Given that one of the aims of this review was to examine participants’ sociodemographic details, the lower age limit of 50 years was therefore selected to maximize inclusivity.

### Intervention

Studies were required to either measure participation in, or provide access to, one or more activities that were described in terms that reflected learning taking place in later life. This included activities described using terms such as lifelong learning/education, adult learning/education, and continuing education in a later life population. Eligible studies included those that investigated engagement in pre-existing LLL activities and those in which the researchers had created a specific learning context or intervention.

### Comparison (or control)

Studies of controlled trials were not excluded.

### Outcome

Studies had to examine the effects of LLL on at least one aspect of health, including physical, cognitive, mental health, or well-being. Both quantitative and qualitative outcomes were considered eligible.

### Information sources

Six electronic databases (CINAHL, MEDLINE, PsycINFO, ERIC, Open Access Theses and Dissertations, and OpenGrey) were systematically searched from inception to August 31, 2024. The PICO framework was used to develop the search strategy, which consisted of three key search concepts: (a) Population: older adults, (b) Intervention: later life learning, and (c) Outcome: health and well-being. The researchers used the PsycINFO thesaurus to identify relevant free-text terms and subject headings for each concept. All the identified search terms were then combined with the Boolean operators OR and AND, and used to search the title, abstract, and subject heading fields of PsycINFO database. This strategy was then adapted to incorporate the appropriate subject headings for each of the other databases (Supplementary Appendix 1, see online supplementary material). The reference lists of all included articles were also manually searched for any additional eligible articles that were not captured in the database search.

### Selection of sources of evidence

Two reviewers (OS and an independent screener) independently screened an initial 10% (*n *= 680) subset of titles and abstracts and then compared the decisions made. Disagreements arose, with the main reviewer (OS) retaining 21 articles while the independent screener retained 30. Through discussion, they clarified the inclusion criteria and agreed that 25 articles should be retained. Screening of the remaining 90% proceeded by the main reviewer (OS). A similar process was used for the full-text screening. This time, both reviewers identified the same 11 articles from the 10% subset as meeting the inclusion criteria. The remaining articles were screened by the main reviewer (OS), with any ambiguities resolved through consultation by a second reviewer (LB).

### Data charting process

A structured spreadsheet was used to guide data extraction from the included studies. The extraction form was divided into three sections: (a) bibliographic data, (b) study aims and characteristics, and (c) specific information relevant to the research questions of this review. The initial data extraction form was piloted by two reviewers (OS and LB) on three studies, and then refined in order to maximize the consistency, accuracy, and thoroughness of data extraction. The main reviewer (OS) then extracted data from the remaining included articles.

### Synthesis of results

For research question 1, all terms used by the authors to describe the broader theoretical context and literature base of LLL were first extracted from each paper. The number of studies reporting each term was then calculated. To capture distinctions in terminology usage, terms with similar meanings (e.g., “lifelong education” and “lifelong learning”) were counted separately rather than grouped. To assess changes in term usage over time, results were organized into weighted intervals that allow for a detailed examination of recent trends while providing a summarized view of earlier years.

To answer research question 2, information that reflected the operationalization and/or measurement of LLL was extracted from each study. That is, for studies assessing the overall effects of participating in a specific LLL activity (such as a pre-existing program, or an intervention provided by the research team), the researchers extracted information about the type(s) of learning activities that were investigated, the setting(s) in which they were undertaken, older learners’ age-inclusion criteria, and duration and frequency of the activities, which reflect how LLL was operationalized. For studies that quantified the amount of LLL activity undertaken by an individual (e.g., by surveying participants about their engagement in LLL activities, or by monitoring levels of attendance/engagement with a particular activity), the researchers extracted details of how this had been measured. Extracted data referring to the measurement and operationalization of LLL were then coded using a coding framework developed by OS and LB. This was done by both authors first reviewing the extracted data and identifying areas of similarity across studies to form potential codes. These initial codes were then iteratively refined during subsequent rounds of coding to ensure clarity, relevance, and comprehensiveness of the codes. A wide variety of codes emerged, which were subsequently organized into distinct dimensions that formed the classification framework. An example of the coding process is provided in Supplementary Appendix 2 (see online supplementary material). In addition, the health-related outcome(s) (e.g., cognitive health, psychological well-being) investigated in each study were extracted. The number of studies examining each outcome was then calculated.

To address research question 3, data relating to the sociodemographic characteristics of participants (i.e., age, gender, education level, marital status, socioeconomic status, and ethnicity) were first extracted from each study. Frequencies of studies that reported each sociodemographic characteristic were then calculated. For age, both the range and unweighted average were computed. Gender, education level, and marital status were quantified as the distribution of (a) female participants, (b) participants with a high level of education (defined as postsecondary nontertiary, and undergraduate/postgraduate tertiary education), and (c) participants who were married or cohabiting, respectively. These distributions were categorized in increments of 10%, ranging from 0%–10% up to 91%–100%.

## Results

**Figure 1.**
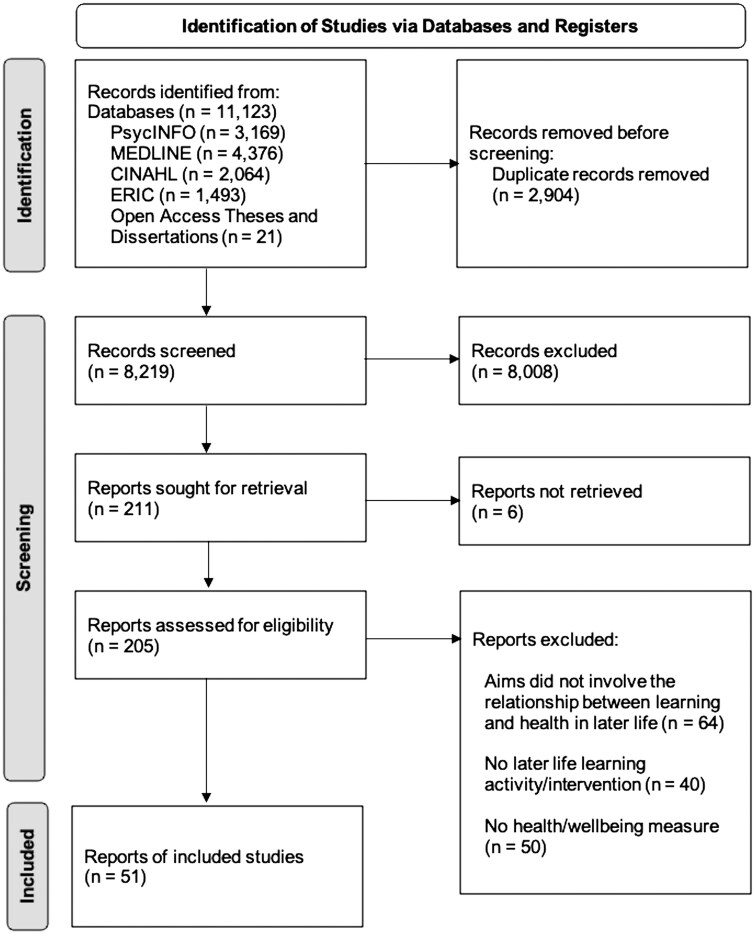
PRISMA flowchart.

A search of the six databases yielded 11,123 records. After removing duplicates, 8,219 records remained for the title and abstract screening (Figure 1). After excluding 8,008 irrelevant records through title and abstract screening, 205 full-text articles were retrieved and screened. Of these articles, 51 met the eligibility criteria for this review. No additional articles were identified through screening citations of the included studies.

### Characteristics of included studies

**Figure 2.**
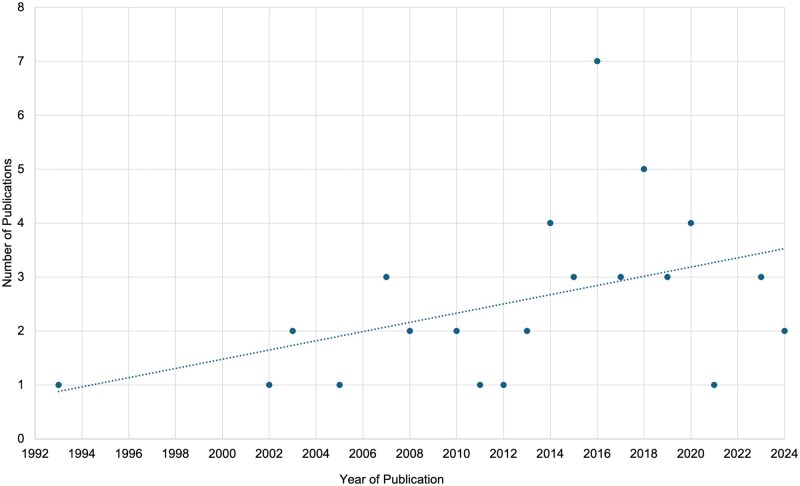
Timeline of LLL and health publications. LLL = later life learning.

The number of LLL and health publications has accelerated since 2012, with approximately three-quarters of the included articles published after that year (Figure 2). As seen in Table 1, most of the included studies originated from two main continental regions: Europe (*n *= 17; 33%) and North America (*n *= 13; 25%). Most studies were quantitative in design (*n *= 31; 61%). Sample sizes varied greatly, ranging from 7 to 3,096 participants.

**Table 1.** Description of characteristics of included studies (*n* = 51).

Characteristic	*n* (%)
**Type of evidence source**	
** Research paper**	49 (96)
** Thesis**	2 (4)
**Region**	
** Europe**	17 (33)
** North America**	13 (25)
** East Asia**	11 (22)
** Australia**	5 (10)
** Western Asia**	3 (6)
** Multiple regions**	1 (2)
** South America**	1 (2)
**Type of study**	
** Observational—investigation of:**	
** a pre-existing, specified learning activity**	23 (45)
** more general engagement in learning activities**	4 (8)
** Interventional—investigation of:**	
** a pre-existing, specified learning activity**	6 (12)
** a learning intervention designed by the researchers**	18 (35)
**Study design**	
** Quantitative**	30 (59)
** Mixed**	11 (22)
** Qualitative**	10 (19)
**Sample size**	
** <10**	2 (4)
** 10–30**	19 (37)
** 31–50**	5 (10)
** 51–100**	4 (8)
** 101–500**	16 (31)
** 501–1000**	3 (6)
** >1000**	2 (4)

Over half of the studies were observational, utilizing quantitative and/or qualitative methods such as surveys or interviews to measure LLL. More specifically, they investigated participation in either (a) a pre-existing, specified learning activity (*n *= 23; 45%) or (b) more general engagement in a range of learning activities (*n *= 4; 8%). An example of the former type is provided by Park et al. (2016), who examined the impact of a university-based learning program for older adults, called Program 60. They employed a cross-sectional design, using a self-report survey to collect data from enrolled older students at the time of the study. Questions included the number of years involved in the program and the number of classes taken. An example of the latter type of study is found in Jenkins and Mostafa (2015), who investigated the longitudinal effects of LLL using data from the English Longitudinal Study of Aging. Here, participants self-reported their participation in various formal and informal learning activities approximately every two years.

Interventional studies were further subdivided into those that provided access to (a) a pre-existing learning activity (*n *= 6; 12%) or (b) a learning activity designed by the research team (*n *= 18; 35%) for a defined period. For instance, Lenehan et al. (2016) explored the impact of university courses, which ran independently to the research study. In contrast, Santini et al. (2020) invited community-dwelling older adults to participate in a 12-month learning program held on farms. A multidisciplinary research team, including the study authors, designed and supervised the activities. In both studies, participants’ attendance was monitored.

A detailed description of the included studies and learning activities is provided in Supplementary Appendix 3 (see online supplementary material).

### Terms used to describe LLL

The researchers identified 25 unique terms used to describe LLL across the included studies (Table 2). The most frequently used term was “Lifelong learning,” which was used in 25 studies, with a notable increase in usage during the periods 2010–2014 (eight studies) and 2015–2019 (eight studies). “Continuing education” was used in nine studies and was consistently mentioned across periods, particularly during 2015–2019 (four studies). Specialized terms with a specific focus on particular activities (such as “Music learning”) or demographics (such as “Later life learning,” “Older adult learning,” and “Third Age education”) appeared in three studies each. In the 2015–2019 period, new terms such as “University study in later life,” “Foreign language learning,” “Leisure education,” and “The learning society” emerged.

**Table 2.** Terms used to describe LLL.

	Number of studies using the term					
Term	Total	1993–2004	2005–2009	2010–2014	2015–2019	2020–2024
**Lifelong learning**	25		3	8	8	6
**Continuing education**	9	1	1	2	4	1
**Lifelong education**	5		2		2	1
**Adult education**	4		1	2	1	
**Health education**	4	1			1	2
**Later life learning**	3			1	2	
**Music learning**	3			1	2	
**Older adult learning**	3		1		2	
**Third Age education**	3	1		1		1
**Adult learning**	2			1		1
**Continuing learning**	2				1	1
**Older adult education**	2		1		1	
**University study in later life**	2				2	
**Computer-use education**	1		1			
**Elder education**	1		1			
**Elder learning**	1				1	
**Foreign language learning**	1				1	
**Internet-use education**	1		1			
**Leisure education**	1				1	
**Nutrition education**	1	1				
**Second language learning**	1				1	
**Senior education**	1			1		
**Technology learning**	1			1		
**The learning society**	1				1	
**Third age learning**	1			1		

*Note*. Column “Total” represents the entire publication period from 1993 to 2024. Years 1993 to 2004 were grouped into a single interval to reflect the early publication phase. From 2005 to 2024, data were summarized in five-year intervals to better capture trends in more recent years. LLL = later life learning.

### Operationalization and/or measurement of LLL, and health-related outcomes

Information was extracted that reflected the operationalization and/or measurement of LLL from each study (Supplementary Appendix 4, see online supplementary material) and quantified according to the classification framework shown in Table 3. Results on the health-related outcomes are presented in Table 4.

**Table 3.** Classification framework for the operationalization and/or measurement of LLL across observational (*n* = 27) and interventional (*n* = 24) studies.

		Observational, *n* (%)			Interventional, *n* (%)		
Dimension	Category [references]	Pre-ex.	Gen. eng.	Overall	Pre-ex.	Design. act.	Overall
**Organizer**	Educational organizations (e.g., Colleges/Universities, adult learning centers) [Åberg, 2016; Borges, 2018; Bužgová et al., 2024; Dias et al., 2017; Ellis, 2018; Escuder-Mollon et al., 2014; Fernández-Ballesteros et al., 2012; Hachem & Vuopala, 2016; Hardy et al., 2017; Hebestreit, 2008; Jenkins & Mostafa, 2015; Lai et al., 2023; Law et al., 2023; Lenehan et al., 2016; Leung & Liu, 2011; Li & Southcott, 2015; Mackowicz & Wnek-Gozdek, 2016; Montoro-Rodriguez & Pinazo, 2005; Narushima et al., 2013; Panayotoff, 1993; Park et al., 2016; Pikhart & Klimova, 2020; Portero & Oliva, 2007; Richeson et al., 2007; Simone & Cesena, 2010; Sloane-Seale & Kops, 2008, 2010; Southcott & Li, 2018; Tam & Chui, 2016; Wang et al., 2018; Wenzel et al., 2024; Zadworna, 2020]	22 (81)	4 (15)	26 (96)	6 (25)		6 (25)
	Non- or for-profit non-educational organizations (e.g., day centers, retirement homes, art galleries) [Åberg, 2016; Jenkins & Mostafa, 2015; Leung & Liu, 2011; Narushima et al., 2013; Pikhart & Klimova, 2020; Sabeti, 2015; Sloane-Seale & Kops, 2008, 2010; Tam & Chui, 2016; Wang et al., 2018]	6 (22)	4 (15)	10 (37)			
	Self-organized (i.e., individuals or groups learning in a variety of ways using a variety of resources) [Åberg, 2016; Sloane-Seale & Kops, 2010; Wang et al., 2018]	1 (4)	2 (7)	3 (11)			
	Research team [Bubbico et al., 2019; Cusack et al., 2003; Díaz-López et al., 2016; Escolar Chua & De Guzman, 2014; Fitzsimmons & Buettner, 2003; Fu et al., 2018; Hsu et al., 2023; Johnson, 2014; Kao & Chang, 2017; MacRitchie et al., 2019; Miller et al., 2002; Perkins & Williamon, 2014; Santini et al., 2020; Seinfeld et al., 2013; Shapira et al., 2007; Shokouhi et al., 2019; Uemura et al., 2021; Valis et al., 2019]					18 (75)	18 (75)
**Target**	Exclusively for older adults	18 (67)	4 (15)	22 (81)	5 (21)	18 (75)	23 (96)
**Audience**	For any older adult [Åberg, 2016; Cusack et al., 2003; Díaz-López et al., 2016; Ellis, 2018; Escuder-Mollon et al., 2014; Fernández-Ballesteros et al., 2012; Hachem & Vuopala, 2016; Hardy et al., 2017; Hebestreit, 2008; Hsu et al., 2023; Jenkins & Mostafa, 2015; Kao & Chang, 2017; Lai et al., 2023; Law et al., 2023; Leung & Liu, 2011; Li & Southcott, 2015; Mackowicz & Wnek-Gozdek, 2016; Montoro-Rodriguez & Pinazo, 2005; Narushima et al., 2013; Panayotoff, 1993; Park et al., 2016; Perkins & Williamon, 2014; Pikhart & Klimova, 2020; Portero & Oliva, 2007; Sabeti, 2015; Santini et al., 2020; Simone & Cesena, 2010; Sloane-Seale & Kops, 2008, 2010; Southcott & Li, 2018; Tam & Chui, 2016; Wang et al., 2018; Wenzel et al., 2024; Zadworna, 2020]	17 (63)	4 (15)	21 (78)	2 (8)	6 (25)	8 (33)
	For older adults who face particular challenges (e.g., family burdens, isolation) [Borges, 2018]	1 (4)		1 (4)			
	For older adults with no particular health issues (e.g., normal blood pressure, no depression) [Bubbico et al., 2019; Bužgová et al., 2024; Dias et al., 2017; Escolar Chua & De Guzman, 2014; Fu et al., 2018; Johnson, 2014; MacRitchie et al., 2019; Seinfeld et al., 2013; Shapira et al., 2007; Shokouhi et al., 2019; Uemura et al., 2021; Valis et al., 2019]				2 (8)	10 (42)	12 (50)
	For older adults with particular health issues (e.g., diabetes, dementia) [Fitzsimmons & Buettner, 2003; Miller et al., 2002; Richeson et al., 2007]				1 (4)	2 (8)	3 (13)
	Mixed-age group [Åberg, 2016; Borges, 2018; Escuder-Mollon et al., 2014; Hardy et al., 2017; Jenkins & Mostafa, 2015; Law et al., 2023; Lenehan et al., 2016; Leung & Liu, 2011; Montoro-Rodriguez & Pinazo, 2005; Narushima et al., 2013; Park et al., 2016; Pikhart & Klimova, 2020; Sloane-Seale & Kops, 2010; Tam & Chui, 2016; Wang et al., 2018]	10 (37)	4 (15)	14 (52)	1 (4)		1 (4)
**Format**	Non-degree courses/programs [Borges, 2018; Bubbico et al., 2019; Bužgová et al., 2024; Cusack et al., 2003; Dias et al., 2017; Díaz-López et al., 2016; Ellis, 2018; Escolar Chua & De Guzman, 2014; Escuder-Mollon et al., 2014; Fernández-Ballesteros et al., 2012; Fitzsimmons & Buettner, 2003; Fu et al., 2018; Hachem & Vuopala, 2016; Hebestreit, 2008; Hsu et al., 2023; Jenkins & Mostafa, 2015; Johnson, 2014; Kao & Chang, 2017; Lai et al., 2023; Law et al., 2023; Leung & Liu, 2011; Li & Southcott, 2015; Mackowicz & Wnek-Gozdek, 2016; Santini et al., 2020; Seinfeld et al., 2013; Shapira et al., 2007; Shokouhi et al., 2019; Simone & Cesena, 2010; Sloane-Seale & Kops, 2008, 2010; Southcott & Li, 2018; Tam & Chui, 2016; Uemura et al., 2021; Valis et al., 2019; Wang et al., 2018; Wenzel et al., 2024; Zadworna, 2020]	20 (74)	4 (15)	24 (89)	5 (21)	18 (75)	23 (96)
	Study/Interest circle [Åberg, 2016; Hachem & Vuopala, 2016; Hebestreit, 2008; Jenkins & Mostafa, 2015; Mackowicz & Wnek-Gozdek, 2016; Portero & Oliva, 2007; Sloane-Seale & Kops, 2008, 2010; Wang et al., 2018; Zadworna, 2020]	7 (26)	3 (11)	10 (37)			
	Workshops/Seminars [Hachem & Vuopala, 2016; Hebestreit, 2008; Jenkins & Mostafa, 2015; Mackowicz & Wnek-Gozdek, 2016; Portero & Oliva, 2007; Sloane-Seale & Kops, 2008, 2010; Tam & Chui, 2016; Wang et al., 2018; Zadworna, 2020]	6 (22)	4 (15)	10 (37)			
	Classes [Jenkins & Mostafa, 2015; Sabeti, 2015; Wang et al., 2018]	1 (4)	2 (7)	3 (11)			
	Degree courses/programs [Hardy et al., 2017; Jenkins & Mostafa, 2015; Lenehan et al., 2016; Tam & Chui, 2016]	1 (4)	2 (7)	3 (11)	1 (4)		1 (4)
	Spontaneous learning that occurs naturally through daily activities such as reading, visiting the museum, watching TV [Sloane-Seale & Kops, 2010; Wang et al., 2018]		2 (7)	2 (7)			
	Outreach programs [Borges, 2018]	1 (4)		1 (4)			
**Content**	Music (e.g., singing, piano learning) [Ellis, 2018; Escuder-Mollon et al., 2014; Fu et al., 2018; Jenkins & Mostafa, 2015; Li & Southcott, 2015; MacRitchie et al., 2019; Narushima et al., 2013; Perkins & Williamon, 2014; Seinfeld et al., 2013; Simone & Cesena, 2010; Sloane-Seale & Kops, 2010; Southcott & Li, 2018; Tam & Chui, 2016]	6 (22)	3 (11)	9 (33)		4 (17)	4 (17)
	Academic topics (e.g., history, literature) [Escuder-Mollon et al., 2014; Fernández-Ballesteros et al., 2012; Hardy et al., 2017; Jenkins & Mostafa, 2015; Lenehan et al., 2016; Montoro-Rodriguez & Pinazo, 2005; Panayotoff, 1993; Park et al., 2016; Simone & Cesena, 2010; Sloane-Seale & Kops, 2010; Tam & Chui, 2016]	5 (19)	3 (11)	8 (30)	3 (13)		3 (13)
	Arts and Crafts (e.g., creative writing, painting) [Escuder-Mollon et al., 2014; Jenkins & Mostafa, 2015; Narushima et al., 2013; Sabeti, 2015; Simone & Cesena, 2010; Sloane-Seale & Kops, 2010; Tam & Chui, 2016]	4 (15)	3 (11)	7 (26)			
	Fitness and Exercise (e.g., sports, dancing) [Borges, 2018; Escolar Chua & De Guzman, 2014; Escuder-Mollon et al., 2014; Jenkins & Mostafa, 2015; Narushima et al., 2013; Sloane-Seale & Kops, 2010; Tam & Chui, 2016]	3 (11)	3 (11)	6 (22)		1 (4)	1 (4)
	Health and well-being education and promotion (e.g., nutrition, dementia) [Borges, 2018; Bužgová et al., 2024; Cusack et al., 2003; Dias et al., 2017; Escolar Chua & De Guzman, 2014; Fitzsimmons & Buettner, 2003; Hsu et al., 2023; Law et al., 2023; Leung & Liu, 2011; Miller et al., 2002; Richeson et al., 2007; Santini et al., 2020; Shokouhi et al., 2019; Sloane-Seale & Kops, 2010; Tam & Chui, 2016; Uemura et al., 2021]	3 (11)	2 (7)	5 (19)	3 (13)	8 (33)	11 (46)
	Languages (e.g., foreign languages, improving existing language skills) [Borges, 2018; Bubbico et al., 2019; Escuder-Mollon et al., 2014; Narushima et al., 2013; Pikhart & Klimova, 2020; Sloane-Seale & Kops, 2010; Valis et al., 2019]	4 (15)	1 (4)	5 (19)		2 (8)	2 (8)
	Technology (e.g., computer use, mobile use) [Borges, 2018; Díaz-López et al., 2016; Escuder-Mollon et al., 2014; Johnson, 2014; Narushima et al., 2013; Shapira et al., 2007; Sloane-Seale & Kops, 2010; Tam & Chui, 2016]	3 (11)	2 (7)	5 (19)		3 (13)	3 (13)
	Leisure (e.g., home repairs, travelling) [Borges, 2018; Kao & Chang, 2017; Sloane-Seale & Kops, 2010; Tam & Chui, 2016]	1 (4)	2 (7)	3 (11)		1 (4)	1 (4)
	Livelihood training (e.g., food preparation, finance management) [Escolar Chua & De Guzman, 2014; Law et al., 2023; Leung & Liu, 2011; Panayotoff, 1993]	2 (7)		2 (7)	1 (4)	1 (4)	2 (8)
	Basic education (e.g., primary, secondary) [Tam & Chui, 2016]		1 (4)	1 (4)			
	Personal development (e.g., self-discovery, public speaking) [Tam & Chui, 2016]		1 (4)	1 (4)			
	Horticulture (e.g., gardening, animal breeding) [Santini et al., 2020]					1 (4)	1 (4)
	Not reported/inferred [Åberg, 2016; Hachem & Vuopala, 2016; Hebestreit, 2008; Lai et al., 2023; Mackowicz & Wnek-Gozdek, 2016; Portero & Oliva, 2007; Sloane-Seale & Kops, 2008; Wang et al., 2018; Wenzel et al., 2024; Zadworna, 2020]	9 (33)	1 (4)	10 (37)			
**Instruction**	Instructor-led learning [Borges, 2018; Bubbico et al., 2019; Bužgová et al., 2024; Cusack et al., 2003; Dias et al., 2017; Díaz-López et al., 2016; Escolar Chua & De Guzman, 2014; Escuder-Mollon et al., 2014; Fernández-Ballesteros et al., 2012; Fitzsimmons & Buettner, 2003; Fu et al., 2018; Hardy et al., 2017; Hsu et al., 2023; Jenkins & Mostafa, 2015; Johnson, 2014; Kao & Chang, 2017; Lai et al., 2023; Law et al., 2023; Lenehan et al., 2016; Leung & Liu, 2011; Li & Southcott, 2015; MacRitchie et al., 2019; Miller et al., 2002; Montoro-Rodriguez & Pinazo, 2005; Narushima et al., 2013; Panayotoff, 1993; Park et al., 2016; Perkins & Williamon, 2014; Pikhart & Klimova, 2020; Richeson et al., 2007; Santini et al., 2020; Seinfeld et al., 2013; Shapira et al., 2007; Shokouhi et al., 2019; Simone & Cesena, 2010; Sloane-Seale & Kops, 2008, 2010; Southcott & Li, 2018; Tam & Chui, 2016; Uemura et al., 2021; Valis et al., 2019; Wang et al., 2018; Wenzel et al., 2024]	15 (56)	4 (15)	19 (70)	6 (25)	18 (75)	24 (100)
**Method**	Peer-led learning [Åberg, 2016; Borges, 2018; Ellis, 2018; Hachem & Vuopala, 2016; Hebestreit, 2008; Jenkins & Mostafa, 2015; Mackowicz & Wnek-Gozdek, 2016; Pikhart & Klimova, 2020; Sabeti, 2015; Sloane-Seale & Kops, 2008, 2010; Tam & Chui, 2016; Wang et al., 2018; Zadworna, 2020]	11 (41)	4 (15)	15 (56)			
	Self-directed learning [Sloane-Seale & Kops, 2010; Wang et al., 2018]		2 (7)	2 (7)			
**Age**	Over 50 years [Cusack et al., 2003; Dias et al., 2017; Hachem & Vuopala, 2016; Hardy et al., 2017; Jenkins & Mostafa, 2015; Li & Southcott, 2015; Perkins & Williamon, 2014; Simone & Cesena, 2010; Southcott & Li, 2018]	5 (19)	1 (4)	6 (22)	1 (4)	2 (8)	3 (13)
	Over 55 years [Díaz-López et al., 2016; Fernández-Ballesteros et al., 2012; Lai et al., 2023; Montoro-Rodriguez & Pinazo, 2005; Pikhart & Klimova, 2020; Portero & Oliva, 2007; Sloane-Seale & Kops, 2010; Valis et al., 2019]	4 (15)	1 (4)	5 (19)	1 (4)	2 (8)	3 (13)
	Over 60 years [Borges, 2018; Bužgová et al., 2024; Escolar Chua & De Guzman, 2014; Fu et al., 2018; Leung & Liu, 2011; Narushima et al., 2013; Park et al., 2016; Richeson et al., 2007; Seinfeld et al., 2013; Zadworna, 2020]	5 (19)		5 (19)	2 (8)	3 (13)	5 (21)
	Over 65 years [Åberg, 2016; Escuder-Mollon et al., 2014; Fitzsimmons & Buettner, 2003; Johnson, 2014; Kao & Chang, 2017; MacRitchie et al., 2019; Miller et al., 2002; Santini et al., 2020; Wenzel et al., 2024]	3 (11)		3 (11)		6 (25)	6 (25)
	Not reported/inferred [Bubbico et al., 2019; Ellis, 2018; Hebestreit, 2008; Hsu et al., 2023; Law et al., 2023; Lenehan et al., 2016; Mackowicz & Wnek-Gozdek, 2016; Panayotoff, 1993; Sabeti, 2015; Shapira et al., 2007; Shokouhi et al., 2019; Sloane-Seale & Kops, 2008; Tam & Chui, 2016; Uemura et al., 2021; Wang et al., 2018]	6 (22)	2 (7)	8 (30)	2 (8)	5 (21)	7 (29)
**Duration**	Short-term	3 (11)	1 (4)	4 (15)	2 (8)	16 (67)	18 (75)
	Less than a month [Lai et al., 2023]	1 (4)		1 (4)			
	1 month [Shokouhi et al., 2019]					1 (4)	1 (4)
	Up to 3 months [Cusack et al., 2003; Fitzsimmons & Buettner, 2003; Fu et al., 2018; Johnson, 2014; Kao & Chang, 2017; MacRitchie et al., 2019; Miller et al., 2002; Perkins & Williamon, 2014; Richeson et al., 2007; Shapira et al., 2007; Valis et al., 2019]				1 (4)	10 (42)	11 (46)
	Up to 6 months [Bubbico et al., 2019; Dias et al., 2017; Díaz-López et al., 2016; Escolar Chua & De Guzman, 2014; Hachem & Vuopala, 2016; Narushima et al., 2013; Seinfeld et al., 2013; Tam & Chui, 2016; Uemura et al., 2021]	2 (7)	1 (4)	3 (11)	1 (4)	5 (21)	6 (25)
	Long-term	9 (33)	2 (7)	11 (41)	3 (13)	1 (4)	4 (17)
	Over 6 months [Borges, 2018; Bužgová et al., 2024; Narushima et al., 2013; Park et al., 2016; Portero & Oliva, 2007]	4 (15)		4 (15)	1 (4)		1 (4)
	1 year [Ellis, 2018; Santini et al., 2020]	1 (4)		1 (4)		1 (4)	1 (4)
	Over 1 year [Jenkins & Mostafa, 2015; Lenehan et al., 2016; Li & Southcott, 2015; Montoro-Rodriguez & Pinazo, 2005; Sloane-Seale & Kops, 2010]	2 (7)	2 (7)	4 (15)	1 (4)		1 (4)
	Over 2 years [Fernández-Ballesteros et al., 2012; Sabeti, 2015; Southcott & Li, 2018]	2 (7)		2 (7)	1 (4)		1 (4)
	Not reported/inferred [Åberg, 2016; Escuder-Mollon et al., 2014; Hardy et al., 2017; Hebestreit, 2008; Hsu et al., 2023; Law et al., 2023; Leung & Liu, 2011; Mackowicz & Wnek-Gozdek, 2016; Panayotoff, 1993; Pikhart & Klimova, 2020; Simone & Cesena, 2010; Sloane-Seale & Kops, 2008; Wang et al., 2018; Wenzel et al., 2024; Zadworna, 2020]	12 (44)	1 (4)	13 (48)	1 (4)	1 (4)	2 (8)
**Frequency**	Once every six months [Wang et al., 2018]		1 (4)	1 (4)			
	Once a month [Borges, 2018; Wang et al., 2018]	1 (4)	1 (4)	2 (7)			
	Once a fortnight [Bužgová et al., 2024; Sabeti, 2015]	1 (4)		1 (4)	1 (4)		1 (4)
	One to two times a week [Bubbico et al., 2019; Dias et al., 2017; Díaz-López et al., 2016; Fitzsimmons & Buettner, 2003; Fu et al., 2018; Johnson, 2014; Kao & Chang, 2017; Li & Southcott, 2015; Miller et al., 2002; Perkins & Williamon, 2014; Richeson et al., 2007; Santini et al., 2020; Seinfeld et al., 2013; Shapira et al., 2007; Southcott & Li, 2018; Uemura et al., 2021; Valis et al., 2019; Wang et al., 2018]	2 (7)	1 (4)	3 (11)	2 (8)	13 (54)	15 (63)
	Several times a week [Borges, 2018; Lai et al., 2023; Wang et al., 2018]	2 (7)	1 (4)	3 (11)			
	Not reported/inferred [Åberg, 2016; Cusack et al., 2003; Ellis, 2018; Escolar Chua & De Guzman, 2014; Escuder-Mollon et al., 2014; Fernández-Ballesteros et al., 2012; Hachem & Vuopala, 2016; Hardy et al., 2017; Hebestreit, 2008; Hsu et al., 2023; Jenkins & Mostafa, 2015; Law et al., 2023; Lenehan et al., 2016; Leung & Liu, 2011; Mackowicz & Wnek-Gozdek, 2016; MacRitchie et al., 2019 Montoro-Rodriguez & Pinazo, 2005; Narushima et al., 2013; Panayotoff, 1993; Park et al., 2016 Pikhart & Klimova, 2020; Portero & Oliva, 2007; Shokouhi et al., 2019; Simone & Cesena, 2010; Sloane-Seale & Kops, 2008, 2010; Tam & Chui, 2016; Wenzel et al., 2024; Zadworna, 2020]	18 (67)	3 (11)	21 (78)	3 (13)	5 (21)	8 (33)

*Note*. For some studies, learning activity was classified into more than one category within a specific dimension. For example, some of the investigated activities encompassed various study topics (e.g., Narushima et al., 2013), while others referred to general participation in any type of formal, non-formal, or informal learning activities (e.g., Wang et al., 2018). Design. act. = learning intervention designed by the researchers; Gen. eng. = more general engagement in learning activities; LLL = later life learning; Pre-ex. = pre-existing, specified learning activity.

**Table 4.** Health-related outcomes reported across observational (*n* = 27) and interventional (*n* = 24) studies.

	Observational *n* (%)			Interventional *n* (%)		
Outcome	Pre-ex.	Gen. eng.	Overall	Pre-ex.	Design. act.	Overall
**General well-being/quality of life**	13 (48)	2 (7)	15 (55)		3 (13)	3 (13)
**Psychological well-being**	6 (22)	2 (7)	8 (29)		5 (21)	5 (21)
**Physical health**	4 (15)	1 (4)	5 (19)	2 (8)	6 (25)	8 (33)
**Cognitive health**	3 (11)		3 (11)	4 (17)	6 (25)	10 (42)
**Physical well-being**	2 (7)	1 (4)	3 (11)			
**Social health**	3 (11)		3 (11)	1 (4)	1 (4)	2 (8)
**Mental health**	2 (7)		2 (7)	5 (21)	4 (17)	9 (38)
**Cognitive well-being**	1 (4)		1 (4)			
**Economic well-being**		1 (4)	1 (4)			
**Health-promoting behaviors**	1 (4)		1 (4)		3 (13)	3 (13)
**Social well-being**		1 (4)	1 (4)			

*Note*. Design. act. = learning intervention designed by the researchers; Gen. eng. = more general engagement in learning activities; Pre-ex. = pre-existing, specified learning activity.

### Which learning activities were included in the studies?

Nearly all observational studies (*n *= 26; 96%) focused on learning activities that were organized by educational organizations, with 81% investigating pre-existing, specified activities, such as university-based educational programs for older adults. Self-organized learning activities (e.g., engaging in volunteer activities) were also reported in three (11%) of observational studies. These were often investigated alongside activities organized by both educational and non-educational organizations, such as University of the Third Age (U3A) courses and recreational classes, respectively. In contrast, most interventional studies (*n *= 18; 75%) assessed the effects of learning interventions designed and organized by the researchers. Examples include a leisure educational program designed to improve leisure attitudes, and a computer and Internet use program aimed to enhance older adults’ technological skills. The remaining interventional studies (*n *= 6; 25%) focused on pre-existing, specified activities organized by educational organizations, such as U3A health courses and university-based educational programs for older adults.

Observational studies primarily involved activities that were exclusively for older adults (*n *= 22; 81%). In addition, 52% of observational studies involved mixed-age group learning activities, allowing intergenerational learning. Notably, all studies in the “general engagement” category (*n *= 4; 15%) were classified under both exclusively for older adults and mixed-age activities due to the broad definitions of LLL. For instance, one study investigated participation in formal and nonformal learning organized by educational and non-educational organizations in the form of courses, workshops, seminars, and lectures. In contrast, nearly all of the interventional studies (*n *= 23; 96%) involved activities that were exclusively for older adults. Half of these (*n *= 12; 50%) targeted specific subgroups of older adults with no particular health issues, while a smaller proportion (*n *= 3; 13%) focused on older adults with specific health conditions, such as diabetes or dementia.

Regarding the “format” dimension, observational studies displayed a broad range of categories, with the majority (*n *= 24; 89%) being nondegree courses/programs. This group included a range of activities that (a) did not lead to a formal degree such as a bachelor’s, master’s, or doctoral degree, (b) were short- or long-term programs/courses designed to provide specialized knowledge, skills, or training in a particular area, and (c) were offered by educational and non-educational organizations or a research team. In the general engagement group (*n *= 4; 15%), formats included activities organized by educational (e.g., U3A seminars) and non-educational (e.g., sports classes) organizations, or self-organized (e.g., reading books, watching TV) by participants. Notably, two of these studies measured and analyzed participation across different formats (Jenkins & Mostafa, 2015; Wang et al., 2018). In interventional studies, only two categories were identified, with nondegree courses/programs being present in nearly all (*n *= 23; 96%), whereas degree courses/programs appeared in only one study (4%).

The “content” dimension showed considerable diversity across studies. In observational studies, the most frequent categories were music (*n *= 9; 33%) and academic topics (*n *= 8; 30%), while the least frequent categories were basic education (*n *= 1; 4%) and personal development (*n *= 1; 4%). Notably, only two studies measured and analyzed participants choices (Sloane-Seale & Kops, 2010; Tam & Chui, 2016). Slightly more than one-third of the observational studies (*n *= 10; 37%) did not report any information related to the content of activities. Conversely, about half of the interventional studies focused on health and well-being (*n *= 11; 46%), followed by music (*n *= 4; 17%). Fitness and exercise (*n *= 1; 4%), horticulture (*n *= 1; 4%), and leisure (*n *= 1; 4%) were the least frequent categories.

For the “instruction” dimension, instructor-led learning emerged as the most predominant category, accounting for 70% of observational studies and 100% of interventional studies. Notably, all observational studies categorized in the “general engagement” group (*n *= 4; 15%) were classified under both instructor- and peer-led activities, reflecting the broad definitions of LLL. These studies investigated participation in various learning formats (e.g., courses, workshops, classes) across diverse settings. Such settings could include, for example, peer-led environments specifically for older adults, such as the U3A, or instructor-led environments such as colleges offering adult education.

### At what age(s) did these learning activities take place?

Age varied in both study types, with 70% of observational studies explicitly defining the age at which LLL occurred by setting age-based inclusion criteria. Approximately one-fifth of observational studies targeted older learners over 50 years (*n *= 6; 22%), while only 11% focused on those over 65 years. About 30% of observational studies did not specify age criteria. Instead, writers used general terms, including “elders,” “older adults,” “older learners,” and “seniors,” to describe the target age.

Similarly, 71% of interventional studies had clearly defined age criteria, with 25% falling into the over 65 years category, followed by over 60 (*n *= 5; 21%), over 55 (*n *= 3; 13%), and over 50 years (*n *= 3; 13%). The remaining 29% described participants with the terms: elderly, older adults, and older people.

### What was the duration and frequency of these learning activities?

Approximately half of the observational studies (*n *= 13; 48%) did not report the duration of participation in LLL. About 41% focused on long-term activities (i.e., over six months), while 11% reported short-term participation (i.e., from a couple of days to up to six months). The frequency of learning activities varied from once every six months to several times a week but, again, most observational studies did not report this detail (*n *= 21; 78%). Notably, only three observational studies measured and analyzed the duration of LLL (Jenkins & Mostafa, 2015; Montoro-Rodriguez & Pinazo, 2005; Narushima et al., 2013), with just one drawing on data from a large-scale longitudinal cohort (Jenkins & Mostafa, 2015). Only one study measured and analyzed frequency (Wang et al., 2018).

In contrast, almost all interventional studies (*n *= 22; 92%) clearly defined the duration of LLL and monitored participants’ attendance. Most of them focused on short-term participation (*n *= 18; 75%). The majority of studies (*n *= 16; 67%) also specified the frequency of learning activities, with nearly all of them concentrating on activities occurring one to two times per week.

### What were the health-related outcomes investigated in the studies?

A wide range of health-related outcomes were examined across the included studies. Observational studies more often investigated the relationship between LLL and general well-being/quality of life (*n *= 15; 55%) and psychological well-being (*n *= 8; 29%), with relatively fewer studies focusing on specific health domains such as physical health (*n *= 5; 19%) or cognitive health (*n *= 3; 11%). In contrast, interventional studies more frequently investigated the effects of LLL on cognitive health (*n *= 10; 42%), mental health (*n *= 9; 38%), and physical health (*n *= 8; 33%). Health-promoting behaviors were rarely assessed, appearing in only 4% of observational studies and 13% of interventional studies.

### Sociodemographic characteristics of older learners

Participants’ sociodemographic characteristics were reported infrequently and not uniformly. Gender was the most frequently reported characteristic across both study types, while ethnicity was reported the least.

### Age

Descriptive statistics on age were inconsistently reported in observational studies (Table 5). Only 48% of these studies provided participants’ mean age; the remaining studies reported age in terms of age groups (nine studies), minimum age (three studies), or did not report age data. In contrast, nearly all interventional studies (*n *= 22; 92%) reported participants’ mean age. The reported mean ages in observational studies ranged from 58 to 71.5 years, with an unweighted average of 65.6 years (Table 6). In contrast, reported mean ages in interventional studies ranged from 59.5 to 83.6 years, with an unweighted average of 70.4 years.

**Table 5.** Reported sociodemographic characteristics of participants across observational (*n* = 27) and interventional (*n* = 24) studies.

	Observational *n* (%)			Interventional *n* (%)		
Characteristic	Pre-ex.	Gen. eng.	Overall	Pre-ex.	Design. act.	Overall
**Mean age**	12 (44)	1 (4)	13 (48)	6 (25)	16 (67)	22 (92)
**Gender**	20 (74)	3 (11)	23 (85)	6 (25)	15 (63)	21 (88)
**Education level**	15 (56)	2 (7)	17 (63)	2 (8)	9 (38)	11 (46)
**Marital status**	9 (33)	3 (11)	12 (44)	3 (13)	8 (33)	11 (46)
**Socioeconomic status**	6 (22)	2 (7)	8 (30)	2 (8)	2 (8)	4 (17)
**Ethnicity**	3 (11)		3 (11)		2 (8)	2 (8)

*Note*. Design. act. = learning intervention designed by the researchers; Gen. eng. = more general engagement in learning activities; Pre-ex. = pre-existing, specified learning activity.

**Table 6.** Range and average of reported mean ages across study types.

	Observational			Interventional		
Mean age	Pre-ex.	Gen. eng.	Overall	Pre-ex.	Design. act.	Overall
**Range**	58–71.5	66.9	58–71.5	59.5–78.8	63.1–83.6	59.5–83.6
**Unweighted average**	65.5	66.9	65.6	67.3	71.6	70.4

*Note*. Design. act. = learning intervention designed by the researchers; Gen. eng. = more general engagement in learning activities; Pre-ex. = pre-existing, specified learning activity.

### Gender

As shown in Table 5, gender data were reported slightly more often in interventional studies (*n *= 21; 88%) compared to observational studies (*n *= 23; 85%). The proportion of female participants across studies ranged from 41% to 100%. In observational studies, the most common level of female representation was in the 61%–70% range, reported by 26% of studies (Table 7). This was followed by 19% of studies reporting female representation within the 71%–80% and 81%–90% ranges, respectively. In interventional studies, the distribution of female participants was more varied. A quarter of studies (*n *= 6; 25%) reported on female participation in the 71%–80% range, followed by 61–70% (*n *= 5; 21%) and 51–60% (*n *= 4; 17%). Only 8% of the interventional studies reported 81%–90% female participants, with only one study (4%) reporting a very high female representation of 91%–100%.

**Table 7.** Distributions of female participants across observational (*n* = 27) and interventional (*n* = 24) studies.

	Observational *n* (%)			Interventional *n* (%)		
Distribution	Pre-ex.	Gen. eng.	Overall	Pre-ex.	Design. act.	Overall
**0%–10%**						
**11%–20%**						
**21%–30%**						
**31%–40%**						
**41%–50%**	1 (4)	1 (4)	2 (7)	2 (8)	1 (4)	3 (13)
**51%–60%**	3 (11)	1 (4)	4 (15)	1 (4)	3 (13)	4 (17)
**61%–70%**	6 (22)	1 (4)	7 (26)	1 (4)	4 (17)	5 (21)
**71%–80%**	5 (19)		5 (19)	1 (4)	5 (21)	6 (25)
**81%–90%**	5 (19)		5 (19)		2 (8)	2 (8)
**91%–100%**				1 (4)		1 (4)
**Not reported**	3 (11)	1 (4)	4 (15)		3 (13)	3 (13)

*Note*. Design. act. = learning intervention designed by the researchers; Gen. eng. = more general engagement in learning activities; Pre-ex. = pre-existing, specified learning activity.

### Education level

More than half of observational studies (*n *= 17; 63%) reported participants’ education level (Table 5), whereas fewer than half of interventional studies included this information (*n *= 11; 46%). The distribution of highly educated participants varied substantially across studies. Among observational studies that reported educational data, 11% each fell into the 31%–40%, 61%–70%, and 91%–100% ranges, indicating relatively high levels of education in these samples (Table 8). Smaller proportions of observational studies (*n *= 2; 7%) each fell within the 21%–30%, 41%–50%, and 51%–60% ranges. One study (4%) reported having 81%–90% highly educated participants. Among interventional studies that reported on education level, 13% had 0%–10% of highly educated participants, while 8% fell within the 31%–40% and 61%–70% ranges. Smaller percentages (4%) of interventional studies reported 11%–20%, 41%–50%, 51%–60%, and 71%–80% of highly educated participants.

**Table 8.** Distributions of (a) participants with a high level of education and (b) participants who were married or cohabiting across observational (*n* = 27) and interventional (*n* = 24) studies.

	Highly educated						Married or cohabiting					
	Observational *n* (%)			Interventional *n* (%)			Observational *n* (%)			Interventional *n* (%)		
Distribution	Pre-ex.	Gen. eng.	Overall	Pre-ex.	Design. act.	Overall	Pre-ex.	Gen. eng.	Overall	Pre-ex.	Design. act.	Overall
**0%–10%**				1 (4)	2 (8)	3 (13)						
**11%–20%**	1 (4)		1 (4)		1 (4)	1 (4)						
**21%–30%**	2 (7)		2 (7)									
**31%–40%**	3 (11)		3 (11)		2 (8)	2 (8)					2 (8)	2 (8)
**41%–50%**		2 (7)	2 (7)		1 (4)	1 (4)	3 (11)		3 (11)			
**51%–60%**	2 (7)		2 (7)		1 (4)	1 (4)	3 (11)		3 (11)	2 (8)	2 (8)	4 (17)
**61%–70%**	3 (11)		3 (11)	1 (4)	1 (4)	2 (8)	1 (4)	2 (7)	3 (11)	1 (4)		1 (4)
**71%–80%**					1 (4)	1 (4)	2 (7)		2 (7)		2 (8)	2 (8)
**81%–90%**	1 (4)		1 (4)					1 (4)	1 (4)		2 (8)	2 (8)
**91%–100%**	3 (11)		3 (11)									
**Not reported**	8 (30)	2 (7)	10 (37)	4 (17)	9 (38)	13 (54)	14 (52)	1 (4)	15 (56)	3 (13)	10 (42)	13 (54)

*Note*. Design. act. = learning intervention designed by the researchers; Gen. eng. = more general engagement in learning activities; Pre-ex. = pre-existing, specified learning activity.

### Marital status

Marital status was reported in a similar proportion across study types (Table 5), with a slight increase in interventional studies (*n *= 11; 46%) compared to observational studies (*n *= 12; 44%). Over half of both observational and interventional studies did not report marital status (Table 8). Among observational studies that reported this data, 11% each were in the 41%–50%, 51%–60%, and 61%–70% ranges. Only 7% of observational studies reported 71%–80% of participants as married or cohabiting, and one study (4%) reported 81%–90%. In interventional studies that reported marital status, 17% indicated that 51%–60% of participants were married or cohabiting. Additional notable distributions included 8% of interventional studies within the 31%–40%, 71%–80%, and 81%–90% ranges, respectively.

### Socioeconomic status (SES)

Participants’ SES was reported inconsistently across studies. Only 30% of observational studies included the information. Among these, five studies classified SES into income categories specific to the study’s year and country, two studies used a scale ranging from “very bad” to “very good,” and one study described all participants as “middle-class.” Similarly, SES was reported in only 17% of interventional studies. Of these, two studies categorized participants by income levels, one study classified SES as either “lower” or “higher,” and one study reported the majority of participants as “middle-class.”

### Ethnicity

Ethnicity was the least reported characteristic across both study types. Only three observational studies reported the information. In the first study, all participants were identified as “White.” The second study provided the following distributions: “White or non-Hispanic” (90.7%), “Hispanic or Latino” (2.8%), “African American” (1.9%), and “Others” (1.9%). Similarly, the third study reported: “White” (91.3%), “Black/African American” (4.7%), “Hispanic/Latino” (1.2%), “Two or more” (1.2%), and “Other” (1.7%). Among the two interventional studies that reported ethnicity, the first study presented the following distributions: “Asian/Pacific Islander” (6.1%), “Caucasian” (87.8%), “Native American” (4.1%), and “Multi-racial” (2.0%). The second study reported: “Afro-Trinidadian” (81%) and “Indo-Trinidadian” (19%).

## Discussion and implications

This scoping review mapped and synthesized information about 51 studies that explored the relationship between LLL and health. The studies were categorized into four groups: observational studies investigating participation in (a) pre-existing, specified learning activities or (b) general engagement in learning, and interventional studies providing access to (c) pre-existing, specified learning activities or (d) researcher-designed learning interventions. The review highlights considerable variation in the operationalization and/or measurement of LLL across the studies. It also provides a classification framework for LLL, encompassing dimensions such as organizer, target audience, format, content, instruction method, age, duration, and frequency, that can be used to describe variation in key study characteristics, and reports details of the health-related outcomes measured in each study. This framework can be used to provide more specific insights into mechanisms that underline links between LLL and health. Notable gaps were identified, including the underrepresentation of male participants and insufficient reporting on education levels, marital status, SES, and ethnicity. These findings provide valuable directions for future research, policy, and practice, and could inform future systematic reviews and meta-analyses exploring the effects of LLL on health.

A diverse and evolving vocabulary was used to describe LLL, with 25 unique terms identified. The term “Lifelong learning” emerged as the most frequently used, reflecting its utility as a comprehensive framework for discussing education across the lifespan. Its sustained popularity, particularly in recent decades, aligns with global policy emphases on LLL as a driver of active aging and social inclusion (Findsen & Formosa, 2016). In contrast, more specialized terms such as “Later life learning,” “Older adult learning,” and “Third Age education” have gained traction in recent years, underscoring a shift toward targeted educational strategies that address the unique needs of older adults (Formosa, 2023).

The emergence of terms such as “Foreign language learning” and “Health education” in the last decades reflects an increasing focus on specific learning and its health impact. For instance, language learning has been associated with cognitive stimulation (Bubbico et al., 2019), while health education can target modifiable health behaviors and specific health outcomes (Uemura et al., 2021). These trends suggest a growing recognition of the potential for tailored LLL initiatives to address particular health challenges, providing a pathway for interventions that combine education with preventative healthcare.

The operationalizations and/or measurements of LLL varied widely across the studies, reflecting the complexity of capturing LLL as a multidimensional construct with diverse organizers, formats, contents, and durations or frequencies of participation. The researchers identified a broad spectrum of learning activities, with programs organized by educational organizations dominating observational studies. However, this may overshadow the contributions of learning organized by non-educational organizations, which are increasingly recognized as critical pathways to learning opportunities (Findsen & Formosa, 2016; Mestheneos & Withnall, 2016). Conversely, interventional studies predominantly investigated researcher-designed learning activities, reflecting a preference for more controlled conditions in which to evaluate outcomes.

The predominance of learning activities tailored exclusively for older adults highlights the need for educational opportunities that address their unique learning needs while fostering peer interaction and support (Formosa, 2023). However, intergenerational learning opportunities, where older adults learn alongside younger generations, were less commonly reported. These activities warrant further exploration as they have the potential to foster social cohesion and reduce age-based stereotypes, contributing to broader health and societal benefits (Field, 2013).

A notable finding was the predominance of nondegree courses/programs in both observational and interventional studies, reflecting the accessibility and flexibility of such formats for older adults (Mestheneos & Withnall, 2016). However, this focus may overlook the potential benefits of degree programs or informal learning contexts, which were less frequently studied. Similarly, the emphasis on instructor-led learning in both study types highlights the importance of structured and guided environments in addressing the educational needs of older adults while fostering engagement and comprehension (Findsen & Formosa, 2016).

The researchers also found substantial diversity in learning content. Activities such as music, academic topics, arts and crafts, languages, and technology reflect specific motivations, preferences, and needs of older learners (Purdie & Boulton-Lewis, 2003; Tyler et al., 2020). In parallel, health and well-being education highlights a growing recognition of the role of health literacy (Nutbeam, 2000) in promoting better outcomes in later life. In addition, fitness and exercise activities align with the broader literature on healthy aging, emphasizing the importance of physical activity for maintaining quality of life in older age (Langhammer et al., 2018). However, many observational studies did not report the content of learning activities. This omission is critical, as different learning contents could involve cognitive (e.g., attention, memory), physical (e.g., intensity, balance, speed), emotional (e.g., managing frustration), and social (e.g., social interaction) dimensions in varying proportions (Illeris, 2002; Jarvis, 2009) that could affect health and well-being in different ways. Understanding these mechanisms requires greater specificity in defining and measuring the content of learning activities.

The variation in age-inclusion criteria across studies indicates a lack of a universal standard for defining “later life” in the context of LLL. Some studies included participants aged 50 years and above, while others focused on individuals aged between 65 and 85 years, contributing to inconsistencies in study populations. Moreover, the inclusion of participants as old as 100 years (e.g., Simone & Cesena, 2010) underscores the broad applicability of LLL across several decades of later life.

The duration and frequency of learning activities were inconsistently reported in observational studies, exposing a critical gap in the literature. Notably, only three of the observational studies included duration as a variable in their analyses (Jenkins & Mostafa, 2015; Montoro-Rodriguez & Pinazo, 2005; Narushima et al., 2013), while just one examined frequency (Wang et al., 2018). This inconsistency complicates cross-study comparisons and limits the ability to determine the “dose” of LLL needed to achieve health benefits. To address this issue, future research should prioritize the standardized reporting of the duration and frequency of learning activities. Although interventional studies provided more detailed reporting on these parameters, their focus on short-term programs raises questions about the long-term sustainability of observed health benefits. Longitudinal studies are urgently needed to investigate whether health benefits persist over time and to identify the optimal balance between duration and frequency.

Across the reviewed studies, investigated health-related outcomes encompassed a broad range of domains, including general well-being/quality of life, psychological well-being, cognitive health, physical health, and, less frequently, social health, health-promoting behaviors, and economic well-being. Observational studies most often assessed general and psychological well-being, whereas interventional studies more frequently targeted cognitive, mental, and physical health outcomes. This diversity in outcome focus reflects the multifaceted nature of LLL as a potential health-promoting activity. By developing a classification framework for LLL and reporting health outcomes, this scoping review provides a foundational evidence base for future systematic reviews and meta-analyses. Such reviews could apply stricter inclusion criteria, aggregate effect sizes, and explore potential moderators (e.g., learning format, content, duration) to not only determine the magnitude of LLL’s impact on specific health domains but also uncover the mechanisms by which different learning approaches contribute to health outcomes.

Sociodemographic characteristics were inconsistently reported across studies. Gender was the most frequently reported characteristic, with women being overrepresented in both study types. This overrepresentation aligns with previous evidence indicating higher participation rates of women in LLL (Jenkins & Mostafa, 2015; Narushima et al., 2013), and may also reflect demographic realities, as women generally have a longer life expectancy than men, leading to a larger population of older women (WHO, 2020). Nevertheless, this finding raises important questions about potential barriers that may hinder male participation in LLL, and their inclusion in related research. Identifying and addressing these barriers is crucial for developing gender-sensitive interventions that promote more equitable participation in LLL.

Education level was the second most frequently reported characteristic, with a higher proportion of participants in observational studies having high education levels. This trend suggests potential recruitment and/or opportunity biases, as individuals with greater educational attainment are likely to have better access to and awareness of LLL activities and greater ability to participate. Furthermore, this disparity highlights how education-related inequalities from a younger age may be perpetuated and even exacerbated by education-related differences in LLL participation. Individuals with lower education may face additional barriers, such as limited prior exposure to formal education, reduced confidence in learning environments, or health issues restricting them from participating in LLL (Farquharson et al., 2024). Notably, over half of the interventional studies did not report participants’ education level, representing a critical gap in the literature. Without this data, it becomes difficult to assess how educational background might influence the effectiveness of LLL interventions, or whether certain groups are systematically excluded from such programs. Future research should address this gap by incorporating education level as a standard variable to ensure more inclusive and equitable LLL opportunities.

Over half of interventional and observational studies did not report marital status, highlighting another important gap in the literature. Among the studies that did, a higher proportion of participants were married or cohabiting. This trend may suggest a potential recruitment bias, as there is evidence that being married is associated with better health and longer life expectancy, while divorce and widowhood negatively affect several health outcomes (Carr et al., 2014; Liu & Waite, 2014). Future studies should account for marital status as a key demographic variable and explore its interaction with other factors to better understand its potential influence on participation in LLL and associated health outcomes.

Lastly, both study types rarely reported SES and ethnicity, underscoring a critical and persistent gap. These omissions are particularly concerning given the influence of SES and cultural factors on access to education and health outcomes (Gómez et al., 2021; McMaughan et al., 2020). Failure to account for these variables limits the generalizability of findings and obscures how LLL impacts diverse populations, especially those from marginalized or underrepresented groups who may encounter significant barriers to participation. Future studies must prioritize more inclusive sampling strategies and comprehensive reporting practices to address this issue. This would improve the generalizability of findings and illuminate how LLL can be adapted to meet the needs of individuals from varied socioeconomic and cultural backgrounds.

### Limitations

While this review provides a comprehensive literature synthesis, some limitations must be acknowledged. First, the inclusion criteria were restricted to studies published in English, which may have excluded relevant research available in other languages. Second, relevant studies may have been missed due to being described by different terms that were not used in the researchers’ search (e.g., specific types of learning activity, such as second language learning; specific populations of older people, such as older immigrants; or specific health outcomes, such as depression or dementia), or by spelling variations not captured in the search (e.g., “life long” and “life-long”). Nevertheless, the search strategy was highly comprehensive, retrieving over 8,000 unique records. The searches of the reference lists of the included papers also did not identify any additional studies, suggesting that the initial search was sufficiently sensitive.

## Conclusion

This scoping review highlights the diverse body of research on LLL and health while introducing a classification framework for LLL. The review also identifies key areas where standardization in reporting could significantly enhance knowledge accumulation and practical application. Despite notable progress in the field, critical gaps remain in the consistent operationalization and measurement of LLL’s multidimensionality. Furthermore, the underrepresentation of specific subgroups of older adults limits the generalizability of findings and obscures how LLL impacts different populations. These gaps hinder a comprehensive understanding of how LLL impacts health. Addressing these challenges is essential for designing effective health interventions and developing evidence-based policies that promote learning and health for all older adults, fostering inclusivity and equity in the benefits of LLL.

## Supplementary Material

gnaf283_Supplementary_Data

## Data Availability

Extraction materials and results are available upon request from Dr. Laura J.E. Brown (ORCID: https://orcid.org/0000-0002-5251-4615). The scoping review protocol was preregistered on the Open Science Framework (https://doi.org/10.17605/OSF.IO/FGVW3).
