# Supplementary material for: Later life learning: what is it, and who is it for? A systematic scoping review of the “learning and health in later life” literature
Source: Gerontologist. 2025 Nov 24;65(12):gnaf283. doi: 10.1093/geront/gnaf283 (PMC12726921; doi:10.1093/geront/gnaf283)
Supplement: gnaf283_Supplementary_Data [file gnaf283_supplementary_data.pdf]

**Supplementary Material for:**

# Later Life Learning: What is it, and Who is it for? A Systematic Scoping Review of the 'Learning and Health in Later Life' Literature

Ourania Sfakianaki, Nick Shryane, Antony Payton, and Laura J.E. Brown

**Appendix 1: Search strategy.**

Full search strategy for PSYCINFO – Ovid

1 (ageing or aging or elderly or "late adulthood" or "later life" or "older adult*" or "old* age" or "old* people" or pension* or retir* or senior).ti,ab,sh.

2 ("later life learning" or "adult education" or "adult learning" or "continuing education" or "continuous education" or "educational programs" or "further education" or learning or "learning activities" or "lifelong education" or "lifelong learning").ti,ab,sh.

3 (health or "quality of life" or wellbeing or "well-being" or "well being").ti,ab,sh.

4 exp health/

5 exp well being/

6 exp quality of life/

7 3 or 4 or 5 or 6

8 1 and 2 and 7

9 limit 8 to (human and english language)

Full search strategy for MEDLINE – Ovid

1 (ageing or aging or elderly or "late adulthood" or "later life" or "older adult*" or "old* age" or "old* people" or pension* or retir* or senior).ti,ab,sh,kw.

2 ("later life learning" or "adult education" or "adult learning" or "continuing education" or "continuous education" or "educational programs" or "further education" or learning or "learning activities" or "lifelong education" or "lifelong learning").ti,ab,sh,kw.

3 (health or "quality of life" or wellbeing or "well-being" or "well being").ti,ab,sh,kw.

4 exp health/

5 exp quality of life/

6 3 or 4 or 5

7 1 and 2 and 6

8 limit 7 to (english language and humans)

Full search strategy for CINAHL – EBSCO

TI ( ageing or aging or elderly or "late adulthood" or "later life" or "older adult*" or "old* age" or "old* people" or pension* or retir* or senior )

AND TI ( "later life learning" or "adult education" or "adult learning" or "continuing education" or "continuous education" or "educational programs" or "further education" or learning or "learning activities" or "lifelong education" or "lifelong learning" )

AND TI ( health or "quality of life" or wellbeing or "well-being" or "well being" )

OR

AB ( ageing or aging or elderly or "late adulthood" or "later life" or "older adult*" or "old* age" or "old* people" or pension* or retir* or senior )

AND AB ( "later life learning" or "adult education" or "adult learning" or "continuing education" or "continuous education" or "educational programs" or "further education" or learning or "learning activities" or "lifelong education" or "lifelong learning" )

AND AB ( health or "quality of life" or wellbeing or "well-being" or "well being" )

OR

MH ( ageing or aging or elderly or "late adulthood" or "later life" or "older adult*" or "old* age" or "old* people" or pension* or retir* or senior )

AND MH ( "later life learning" or "adult education" or "adult learning" or "continuing education" or "continuous education" or "educational programs" or "further education" or learning or "learning activities" or "lifelong education" or "lifelong learning" )

AND MH ( health or "quality of life" or wellbeing or "well-being" or "well being" )

Limiters - Human; Language: English

Full search strategy for ERIC – EBSCO

TI ( ageing or aging or elderly or "late adulthood" or "later life" or "older adult*" or "old* age" or "old* people" or pension* or retir* or senior ) AND TI ( "later life learning" or "adult education" or "adult learning" or "continuing education" or "continuous education" or "educational

programs" or "further education" or learning  or "learning activities" or "lifelong education"  or "lifelong learning" ) AND TI ( health  or "quality of life" or wellbeing  or "well-being"  or "well being"  or MH health+ or MH "well being"+ or MH "quality of life"+ )

OR

AB ( ageing or aging or elderly or "late adulthood"  or "later life" or "older adult*" or "old* age" or "old* people" or pension* or retir* or senior ) AND AB ( "later life learning" or "adult education" or "adult learning" or "continuing education" or "continuous education"  or "educational programs" or "further education" or learning  or "learning activities" or "lifelong education"  or "lifelong learning" ) AND AB ( health  or "quality of life" or wellbeing  or "well-being"  or "well being"  or MH health+ or MH "well being"+ or MH "quality of life"+ )

OR

SU ( ageing or aging or elderly or "late adulthood"  or "later life" or "older adult*" or "old* age" or "old* people" or pension* or retir* or senior ) AND SU ( "later life learning" or "adult education" or "adult learning" or "continuing education" or "continuous education"  or "educational programs" or "further education" or learning  or "learning activities" or "lifelong education"  or "lifelong learning" ) AND SU ( health  or "quality of life" or wellbeing  or "well-being"  or "well being"  or MH health+ or MH "well being"+ or MH "quality of life"+ )

**Appendix 2: Figure S1. Example of how the coding framework was developed, using extracted data from Santini et al. (2020) to illustrate each step of the process from data extraction to dimension development.**

**1. Extracted information**

This pilot study was based on 12-month green care educational program delivered by six farms for the enhancement of four Active Aging determinants (physical health, psychological well-being, social engagement, and lifelong learning among frail older adults living in the community). (p. 1241)

The only inclusion criteria for older adults were that they had to be aged 65 years and above and living in the community or attending a day care center. (p. 1241)

The program was delivered over 50 full days (ca. 6 hr a day) of training on the farms for a period of 12 months. (p. 1242)

The farms set up multidisciplinary teams incorporating experts of social sciences, agriculture, and medicine, such as, psychologists, occupational therapists, and nutritionists. (p. 1242)

Activities were delivered in group sessions, and each group had one or two tutors. (p. 1242)

Horticulture, Education on agricultural theory and practice, Gym and other related activities for mental and physical well-being (p. 1243)

Santini et al. (2020)

**2. Initial thoughts and coding**

- Pilot study
- Organized and delivered by a multidisciplinary research team
- Non-formal, farm-based, educational program
- Enrolment criterion 1: Older adults aged 65 years and above
- Enrolment criterion 2: Older adults living in the community or attending a day care center
- Activities promoting mental and physical well-being
- Activities involving horticulture and agriculture
- Group sessions with tutors
- 12-month duration
- 50 full days (6 hr a day)

**3. Refined codes**

- Research team
- Exclusively for older adults
- For any older adult
- Non-degree courses/programs
- Health and wellbeing education and promotion
- Horticulture
- Instructor-led
- Over 65 years
- Long-term
- 1 year
- 1-2 times a week

**4. Dimension development**

- Organizer
- Target audience
- Format
- Content
- Instruction method
- Age
- Duration
- Frequency

**Reference**

Santini, S., Piccinini, F., & Gagliardi, C. (2020). Can a green care informal learning program foster Active Aging in older adults? Results from a qualitative pilot study in central Italy. *Journal of Applied Gerontology: The Official Journal of the Southern Gerontological Society*, *39*(11), 1240–1249. https://doi.org/10.1177/0733464819883769

**Appendix 3: Table S1. Description of the included studies.**

| Reference | Country | Design | | Sample size | Mean age | Female % | LLL Activity | LLL Location | LLL Duration & Frequency |
|---|---|---|---|---|---|---|---|---|---|
| **Observational - investigation of a pre-existing, specified learning activity** | | | | | | | | | |
| Åberg (2016) | Sweden | Quantitative | Survey | 1,499 | | 70.0% | Swedish study circles (various topics) | Various educational and non-educational settings | |
| Borges (2018) | Brazil and Canada | Qualitative | Individual interviews, Focus group, Observations | 14 | 71.20 | 71.0% | Educational program for older adults (various courses) | College/University | Over 6 months |
| Ellis (2018) | Australia | Mixed | Focus group, Observation, Questionnaire | 21 | | 57.1% | Music learning - Ukulele | U3A | 1 year |
| Escuder-Mollon et al. (2014) | Italy, Spain, Poland | Quantitative | Survey | 100 | 65.00 | 66.0% | Educational program for older adults (various courses) | College/University | |
| Hachem & Vuopala (2016) | Lebanon | Qualitative | Questionnaire | 461 | | 83.0% | U3A (various courses) | U3A | One term |
| Hardy et al. (2017) | Australia | Qualitative | Individual interviews | 15 | 61.00 | 66.7% | Tertiary education (various courses) | College/University | |
| Hebestreit (2008) | Australia | Quantitative | Survey | 627 | 71.50 | | U3A (various courses) | U3A | |
| Lai et al. (2023) | Hong Kong | Qualitative | Individual interviews | 32 | | 78.0% | Educational program for older adults (various courses) - MiniU | College/University | 2 weeks with 10 full days (about 6 hours per day) |
| Law et al. (2023) | Hong Kong | Mixed | Individual interviews, Questionnaire | 20 | | 80.0% | Educational program for adults (various courses) - The Diploma in Active Ageing (DAA) | College/University | |
| Leung & Liu (2011) | Hong Kong | Quantitative | Survey | 109 | | 90.0% | Educational program for adult women (various courses) - The Capacity Building Mileage Programme | Various educational and non-educational settings | |
| Li & Southcott (2015) | China | Qualitative | Individual interviews, Observations | 16 | 62.10 | 75.0% | Music learning - Keyboard | Community-focused educational center for older adults | Weekly 2hr lessons |

| | | | | | | | | | |
|---|---|---|---|---|---|---|---|---|---|
| Mackowicz & Wnek-Gozdek (2016) | Poland | Qualitative | Individual interviews | 11 | | | U3A (various courses) | U3A | |
| Montoro-Rodriguez & Pinazo (2005) | Spain | Quantitative | Survey | 212 | 62.00 | 55.7% | Intergenerational educational program for older adults (various courses) - Nau Gran | College/University | Program exposure refers to the length of participation in the program, and it is measured using their academic status as sophomore (0) and junior (1) students at the university |
| Narushima et al. (2013) | Canada | Quantitative | Survey | 699 | 69.21 | 73.5% | Educational programs for adults (various courses) | Various settings including high schools, adult learning centres, community centres, and retirement homes | Different rates of participation: up to 1.5 years, up to 4 years, over 4 years |
| Park et al. (2016) | USA | Quantitative | Survey | 107 | 67.97 | 46.7% | Educational program for older adults (various courses) - Program 60 | College/University | Participation was defined as the number of years older adults had been involved in the program. |
| Pikhart & Klimova (2020) | Czech Republic | Quantitative | Survey | 105 | | 65.0% | Foreign language learning - English, German | U3A & company-organised | |
| Portero & Oliva (2007) | Spain | Quantitative | Survey | 147 | 58.00 | 62.5% | U3A (various courses) | U3A | One academic year |
| Sabeti (2015) | UK | Qualitative | Individual interviews, Observations | 14 | | | Creative writing classes | Art gallery | |
| Simone & Cesena (2010) | USA | Quantitative | Survey | 116 | 68.00 | 60.0% | Educational programs for older adults (various courses) | College/University & Adult educational centre | |
| Sloane-Seale & Kops (2008) | Canada | Qualitative | Focus group | 38 | | 84.2% | Lifelong learning programs for older adults | Various educational and non-educational settings | |
| Southcott & Li (2018) | China | Qualitative | Individual interviews, Observations | 13 | 61.40 | 61.5% | Music learning - Singing | Community-focused educational center for older adults | Over three years. Weekly. |
| Wenzel et al. (2024) | USA | Quantitative | Survey | 344 | | 81.9% | Educational program for older adults (various courses) | College/University | |
| Zadworna (2020) | Poland | Quantitative | Survey | 130 | 68.80 | 87.7% | U3A (various courses) | U3A | |

**Observational - investigation of more general engagement in learning activities**

| Reference | Country | Design | | Sample size | Mean age | Female % | LLL Activity | LLL Location | LLL Duration & Frequency |
|---|---|---|---|---|---|---|---|---|---|
| Jenkins & Mostafa (2015) | UK | Quantitative | Longitudinal cohort survey | 3096 | | | Formal and informal learning | Various educational and non-educational settings | Longitudinal survey - Up to 7 years |
| Sloane-Seale & Kops (2010) | Canada | Quantitative | Survey | 221 | | 59.5% | Formal and informal learning | Various educational and non-educational settings | Up to 2 years since the data collection |
| Tam & Chui (2016) | Hong Kong | Quantitative | Survey | 283 | | 62.9% | Formal and non-formal learning | Various educational and non-educational settings | Up to 6 months since the data collection |
| Wang et al. (2018) | China | Mixed | Focus-group, Questionnaire | 579 | 66.94 | 49.9% | Formal, non-formal, and informal learning | Various educational and non-educational settings | They measured the frequency of engagement in learning activities using a 5-point Likert scale ranging from 1 (never), 2 (once half a year), 3 (monthly), 4 (weekly), and 5 (daily). |

**Interventional - investigation of a pre-existing, specified learning activity**

| Reference | Country | Design | | Sample size | Mean age | Female % | LLL Activity | LLL Location | LLL Duration & Frequency |
|---|---|---|---|---|---|---|---|---|---|
| Bužgová et al. (2024) | Czech Republic | Quantitative | Quasi-experimental design - one-group intervention | 121 | 71.45 | 90.9% | Health education and promotion - Living More Healthily and Actively | U3A | One academic year. The lectures were run once every two weeks for 90 minutes |
| Dias et al. (2017) | Brazil | Quantitative | Quasi-experimental design | 13 | 66.40 | 76.9% | Health education and promotion | U3A | The program lasted 4 months. Sessions were held once every week and were lasting for an hour. |
| Fernández-Ballesteros et al. (2012) | Spain | Quantitative | Quasi-experimental design | 56 | 60.89 | 50.0% | Educational program for older adults (various courses) - University Program for Older Adults (PUMA) | College/University | 3 academic years with a total of 450 teaching hours |
| Lenehan et al. (2016) | Australia | Quantitative | Quasi-experimental design | 359 | 59.48 | 69.5% | Tertiary education (various courses) | College/University | Minimum participation 12 months (full or part-time university study) |

| Reference | Country | Design | | Sample size | Mean age | Female % | LLL Activity | LLL Location | LLL Duration & Frequency |
|---|---|---|---|---|---|---|---|---|---|
| Panayotoff (1993) | USA | Quantitative | Quasi-experimental design | 114 | 67.00 | 55.0% | Educational programs for older adults (various courses) | College/University | |
| Richeson et al. (2007) | USA | Mixed | Quasi-experimental design - one-group intervention | 12 | 78.80 | 50.0% | Health education and promotion - Health Promotion for the Mind, Body, and Spirit | College/University | The course lasted 13 weeks, and it was held on Fridays from 9:30 to 11:30 a.m. |
| **Interventional - investigation of a learning intervention designed by the researchers** | | | | | | | | | |
| **Reference** | **Country** | **Design** | | **Sample size** | **Mean age** | **Female %** | **LLL Activity** | **LLL Location** | **LLL Duration & Frequency** |
| Bubbico et al. (2019) | Italy | Quantitative | Randomized control design | 14 | 69.50 | 85.7% | Foreign language learning - English | N/A | The program lasted 4 months (16 weekly sessions). Each training week consisted of a 1 h and a half classroom session |
| Cusack et al. (2003) | Canada | Quantitative | Quasi-experimental design - one-group intervention | 18 | 68.00 | | Health education and promotion - Mental Fitness for Life Program | N/A | 8 weeks |
| Díaz-López et al. (2016) | Spain | Quantitative | Randomized control design | 112 | 69.38 | 71.4% | Information and communication technology course | Community centers | 4 months. 2 weekly sessions of an hour and a half each. |
| Escolar Chua & De Guzman (2014) | Philippines | Quantitative | Randomized control design | 25 | | 60.0% | Educational programs for older adults (various courses) | Community centers | The program lasted 4 months |
| Fitzsimmons & Buettner (2003) | USA | Mixed | Quasi-experimental design - one-group intervention | 7 | 77.90 | 71.4% | Health education and promotion - Health Promotion for the Mind, Body, and Spirit | College/University | 10 weeks. Class was held on Wednesdays from 10:00 a.m. to noon. |
| Fu et al. (2018) | USA | Quantitative | Quasi-experimental design - one-group intervention | 49 | 83.60 | 79.6% | Music learning - Singing | N/A | 3 months. Weekly 75-min sessions |
| Hsu et al. (2023) | Taiwan | Mixed | Quasi-experimental design - one- | 221 | 71.10 | 87.0% | Health education and promotion - KABAN! | Various outdoor venues | |

| | | | | | | | | | |
|---|---|---|---|---|---|---|---|---|---|
| | | | group intervention | | | | | | |
| Johnson (2014) | The Republic of Trinidad and Tobago | Mixed | Quasi-experimental design - one-group intervention | 16 | 68.88 | 68.7% | Digital communication technologies course | Community centers | 6 weeks. Two hours each week |
| Kao & Chang (2017) | Taiwan | Quantitative | Randomized control design | 20 | 69.40 | 55.0% | Leisure education program | College/University | 3 months. Twice per week, 2 hrs in each unit |
| MacRitchie et al. (2020) | Australia | Mixed | Randomized control design | 8 | 71.40 | 75.0% | Music learning - Piano | N/A | The 10-week training program consisted of 10 lessons, each of 60 minutes duration |
| Miller et al. (2002) | USA | Quantitative | Randomized control design | 45 | 72.10 | 64.4% | Health education and promotion - nutrition | N/A | 10 weeks. Weekly 1.5 to 2 hours sessions |
| Perkins & Williamon (2014) | UK | Mixed | Quasi-experimental design | 68 | 68.00 | 75.0% | Music learning - Various musical instruments | College/University | 10 weeks. Weekly lessons 1 to 2 hrs |
| Santini et al. (2020) | Italy | Qualitative | Quasi-experimental design - one-group intervention | 90 | | 64.4% | Educational program for older adults (various courses) - Green Care Program | Farms | The program was delivered over 50 full days (about 6 hr a day) of training on the farms for a period of 12 months. |
| Seinfeld et al. (2013) | Spain | Quantitative | Quasi-experimental design | 13 | 69.30 | | Music learning - Piano | Community centers | 4 months. One and a half hr weekly lessons |
| Shapira et al. (2007) | Israel | Mixed | Quasi-experimental design | 22 | 80.25 | 59.1% | Computer operation and Internet use course | N/A | 15 weeks. One or two lessons per week, each approximately 60 minutes long |
| Shokouhi et al. (2019) | Iran | Quantitative | Randomized control design | 43 | 63.12 | 41.9% | Health education and promotion - oral health | Dental clinic | One month |
| Uemura et al. (2021) | Japan | Quantitative | Randomized control design | 30 | 74.00 | 66.7% | Health education and promotion - arterial stiffness | N/A | The program lasted 24 weeks. It was consisted of weekly 90-min sessions. |
| Valis et al. (2019) | Czech Republic | Quantitative | Randomized control design | 20 | 69.80 | | Foreign language learning - English | N/A | 12 weeks. 3 lessons of 45-min duration once a week, every Wednesday. |

*Note*. LLL = Later life learning; U3A = University of the Third Age.

**Appendix 4: Table S2. Operationalization and/or measurement of LLL, and investigated health-related outcomes in the included studies**

| Reference | Design | | Operationalization and/or measurement of LLL | Health-related outcomes | Key findings |
|---|---|---|---|---|---|
| **Observational - investigation of a pre-existing, specified learning activity** | | | | | |
| Åberg (2016) | Quantitative | Survey | LLL was defined as enrolment in Swedish study circles. A study circle is a group of individuals (no age restrictions) who come together to study a common topic/theme. It can be self-organized or organized by other organizations. Study circles operate on principles of equality and democracy. All participants have an equal opportunity to contribute, share their knowledge, and influence the direction of the group's learning. Study circles can cover a wide range of subjects. The topics are often chosen based on the interests and needs of the participants. This study used data from a nationwide survey of individuals who participated in a study circle in 2012 and who were 13 years old or older at the time. Specifically, they selected data for people aged over 65. No information about the duration was recorded; participation was defined simply on the basis of being listed on the Study Associations' Activity Register. | General wellbeing/Quality of life | The study highlighted the significant role of social interaction and fellowship within the Swedish study circles, finding that these aspects are as important as the acquisition of new knowledge and skills for fostering a sense of wellbeing and combating social isolation. |
| Borges (2018) | Qualitative | Interviews, focus groups, and observations | LLL was defined as enrolment in educational programs for older adults at any of the following Universities: (1) Lifelong Learning Centre, University of Regina, Canada, and (2) Universidade da Melhor Idade, Universidade Católica Dom Bosco, Brazil. For the purposes of this study, the age-based inclusion criterion for participants was over 60 years and they needed to have been registered and actively participating in one of these two educational programs for more than six months. Universidade da Melhor Idade, Universidade Católica Dom Bosco (Brazil): program designed for people 50 years and older. The program is free (apart from a one-off registration fee) and offers a wide range of courses, such as language classes, fitness classes, sports, aquasize, nutritional classes, psychosocial aspects of ageing, healthy ageing, computer classes, field trips, and political issues. All activities are thought out and planned for the older adult population. These older adults actively participate in the decision of the creation of the curriculum, suggesting what they think is important and what they want to learn, in order to improve their quality of life. Classes are led by professors/instructors, and | General wellbeing/Quality of life | Four themes emerged through the participants' narratives, photos, diaries and observations regarding the role that educational programs for older adults play in promoting healthy living and wellbeing: Fighting Social Isolation, Stimulating Cognitive and Mental State of Wellbeing, Fostering Physical Health, and Promoting (Dis)Ageism. |

| | | | | | |
|---|---|---|---|---|---|
| | | | they take place in the University from Monday to Friday, beginning at 1:30 p.m. until 5 p.m. Participants have to attend at least three days a week, and can choose which courses they want to take, including three suggested classes: sports/fitness (focusing on physical health and well-being); psychosocial aspects of aging (focusing on better understanding the changes that happens with age); and healthy aging (focusing on health promotion).<br>Lifelong Learning Centre, University of Regina (Canada):<br>to participate in this program, older adults do not need any prior level of education. There are no exams, papers or assignments. Each participant is responsible for the course fee which can vary from $70 to $200. The older adults participate in every aspect of the program. At the end of every course offered, there is a course evaluation form asking for suggestions or feedback. The classes are led by instructors and are student-centred. Outreach programs are also offered, including the Aboriginal Grandmothers Caring for Grandchildren Support Network (AGCGSN). This group was created because the Indigenous grandmothers were feeling isolated and had challenges raising their grandchildren who were seized by the Child and Family Services department. The Lifelong Learning Program provides the infrastructure such as a meeting space, printing and computer services, and administrative support; and the program participants organize and run the meetings in a "talking circle" format, similar to a focus group. | | |
| Ellis (2018) | Mixed | Questionnaires, focus group, and observations | LLL was defined as enrolment in a music learning program (learning to play the ukulele) at the U3A. No specific age limits for 'later life' were defined. Participants were described as 'older learners', mostly aged 65 and over. The learning program lasted for a year, although no details regarding the frequency of participation were documented. | General wellbeing/Quality of life | The study demonstrated that participation in music learning in later life fostered enjoyment, social connection, a sense of achievement, and even physical improvements despite initial challenges. |
| Escuder-Mollon et al. (2014) | Quantitative | Survey | LLL was defined as enrolment to educational classes at three specific institutions: (1) the Senior Citizens' University at Jaume I University in Spain, (2) the Universutà delle LiberEtà del FVG in Italy, and (3) Akademia im. Jana Dlugosza w Czestochowie (AJD) in Poland, undertaken by citizens over 65 or retired.<br>The subjects and activities of each institution were as follows:<br>Spain: humanities, psychology, society, history, arts, information and communication technology, language courses, museum visits, cultural trips, drama, choir, trekking, radio, learners' magazine.<br>Italy: Broad culture courses, foreign languages, information and communication technology courses, courses for physical wellbeing, gymnastics, dancing, artistic laboratory, handicrafts, ceramics, sewing. | General wellbeing/Quality of life | Findings suggested that educational pursuits in later life contribute to physiological wellbeing, enjoyment, and personal adaptation among older adults. The study highlighted that while basic needs are crucial, a key factor in improving older adults' quality of life through education is the teacher's social skills and ability to transmit knowledge, even more so than specific course content or pedagogical approaches. |

| | | | | | |
|---|---|---|---|---|---|
| | | | Poland: ordinary subjects (broad culture), conferences, information and communication technology courses, activities done in the open air or related to physical well-being.<br>The survey, which was conducted as part of the European project QEduSen, did not record the duration or frequency of participation. | | |
| Hachem & Vuopala (2016) | Qualitative | Survey | LLL was defined as enrolment in the U3A, and older students were defined as individuals aged 50 years and older. Participants were purposively recruited from two cohorts in a U3A in Lebanon. One in the fall term and another in the following spring term of the same academic year. The curriculum consisted of a wide range of topics from health, culture, humanities, and economics to basic sciences and arts. | Cognitive health, Social health, Psychological wellbeing | Findings indicate that older adults experience cognitive, social, and psychological benefits from their learning, with cognitive gains being the most frequently reported. |
| Hardy et al. (2017) | Qualitative | Interviews | LLL was defined as enrolment in higher education courses (i.e., Tertiary preparation pathway, Degree, Masters, PhD) after the age of 50. No information about the courses or duration was recorded. | Cognitive health, Social health | The majority of participants in this study reported that the main benefit of university education was to their cognitive health. |
| Hebestreit (2008) | Quantitative | Survey | LLL was defined as enrolment in the U3A. No specific age limits for 'later life' were defined. Participants were described as 'older adults', with mean ages of 70.3 years for females and 73.3 for males. No information about the courses or duration was recorded. | General wellbeing/Quality of life | Findings indicated that older learners were satisfied with their U3A experiences, which had contributed in various areas of their lives, leading to personal, mental, social, and physical enhancement. |
| Lai et al. (2023) | Qualitative | Interviews | LLL was defined as enrolment in the MiniU program, an ongoing annual summer program organised by the Institute of Active Aging at the Hong Kong Polytechnic University since 2007.<br>The purpose of this program is to make good use of the university's built environment and space during the summer break to provide an experience of university life for older people aged 55 and above. Each summer, the MiniU offers a two-week program with 10 full days (about 6 hours per day) of classes on a variety of practical and leisure subjects. It provides an overall university experience for participants including a learning experience at a university campus, a one-night stay in student accommodations, and a graduation ceremony. | Physical wellbeing, Psychological wellbeing | Eight participants stated that learning health-related knowledge helped them improve their physical health condition. Moreover, participating in activities and learning new things motivated them to join more outdoor activities, which helped improve their physical and mental health. Six participants expressed that participating in MiniU encouraged them to embrace more positive emotions including happiness, sense of purpose, satisfaction, and self-fulfilment. |
| Law et al. (2023) | Mixed | Questionnaires and interviews | LLL was defined as enrolment in a lifelong educational program, the Diploma in Active Ageing (DAA), at the School of Professional Education and Executive Development of The Hong Kong Polytechnic University (PolyU SPEED).<br>No specific age limits for 'later life' were defined. Participants were described as 'older adults', with ages between 50 and 79 years.<br>DAA aims to equip students with transferable skills and knowledge, such as nutrition and physical fitness, psychology of ageing and dementia care, for enhancement of the daily living activities of active older adults. | Physical health, Mental health, Social health | The results showed that older adults who received both informal and formal learning opportunities experienced positive changes to their physical, mental, and social health. |

| | | | | | |
|---|---|---|---|---|---|
| | | | DAA offers subjects covering ageing-related knowledge, holistic health wellness and financial planning. Students who have completed 10 compulsory subjects with satisfactory results are awarded a diploma, while those who have completed any three subjects receive a certificate. No information about the duration was recorded. | | |
| Leung & Liu (2011) | Quantitative | Survey | LLL was defined as enrolment in a lifelong educational program for adult women - The Capacity Building Mileage Programme (CBMP). The CBMP was developed by the Women's Commission of the Hong Kong Special Administrative Region Government in partnership with the Open University of Hong Kong, Commercial Radio of Hong Kong, and some nongovernment Hong Kong organisations. The Program provides adult learners—especially women—with unconventional, good quality, specially designed non-credit courses delivered via radio broadcasts as well as by face-to face teaching modes. Courses offered in the program include managing interpersonal relationships, finance management, health, and other practical issues relating to one's daily life. The present study involved all the CBMP students (age range: 18–78). Later life was defined as participants aged 60 and above. No information on the duration/frequency of participation was recorded. | General wellbeing/Quality of life | Findings revealed that the patterns of participation in learning established a positive association with quality of life, but a negative correlation with older learners' psychosomatic complaints. It also found that the more respondents enrolled in courses, the more somatic complaints they suffered. Multiple regression analysis models indicated that the determinants of good quality of life in older adult learners aged 60 and over were good self-efficacy and the continuation of study after graduation. |
| Li & Southcott (2015) | Qualitative | Interviews and observations | LLL was defined as the enrolment of individuals aged 50 and older in keyboard lessons (basic, intensive, and master-level classes) at the Tangshan Older People University (TOPU), China. Regarding the duration of participation, older students had been attending the classes for between 1 and 4 years. The lessons were held weekly and lasted for two hours. | General wellbeing/Quality of life | This research used interpretative phenomenological analysis to understand the participants' motivations and the impact of music education on their later lives. Two broad wellbeing-related themes were identified from the data: emotional wellbeing and physical wellbeing. |
| Mackowicz & Wnek-Gozdek (2016) | Qualitative | Interviews | LLL was defined as enrolment in the U3A at the Pedagogical University of Cracow, Poland, which offers lectures and runs classes, workshops, courses, and science clubs. No specific age limits for 'later life' were defined. Participants were described as 'seniors'. No information about the activities or the duration of attendance was recorded. | General wellbeing/Quality of life | Analysis revealed that participation in U3A classes was beneficial for the quality of older adults' lives, preventing isolation and resulting in positive changes on both individual and social dimensions, generating long-term benefits. |
| Montoro-Rodriguez & Pinazo (2005) | Quantitative | Survey | LLL was defined as enrolment in the intergenerational program "Nau Gran" at the University of Valencia, Spain. This program was initiated in 1999-2000 and allows older adults, over the age of 55, to enrol in both core-mandated subjects and electives courses offered by the University. The program offers a variety of courses such as Psychology, Art History, Humanities, Geography-History, Health Sciences, Social Sciences, and Sciences. The main objectives of the program are the integration of older adults into university life, the development of positive attitudes toward ageing, the improvement of | Psychological wellbeing | The findings revealed that longer participation in the learning programme significantly increased social integration and personal growth among older adults, although no immediate significant impact on psychological wellbeing measures was observed. |

| | | | | | |
|---|---|---|---|---|---|
| | | | intergenerational relations, and the enhancement of the quality of life for older adults. The duration of participation in the program was measured using older students' academic status as sophomore (0) and junior (1) students at the university. | | |
| Narushima et al. (2013) | Quantitative | Survey | LLL was defined as enrolment in a public non-formal continuing education program in Ontario, Canada. The program has been run by a local school board in partnership with various community facilities including high schools, adult learning centres, community centres, and retirement homes. The program offers non-credit and nonvocational general interest courses in two streams: daytime courses designated for people over 60, and courses mostly offered in the evening and open to anyone over 18 years old. The contents are generally classified into four subject areas: (a) arts and crafts, (b) fitness and exercise, (c) music and dance, and (d) language and computers and other practical skills. In this study, researchers targeted older learners aged over 60, who were enrolled in either daytime or evening courses during the fall semester of 2010. The survey included a question regarding the duration of taking the current course or subject. The duration was further split into three groups: 4 to 18 months-short duration, 19 to 48 months-mild duration, and 49 months and longer-long duration. Those who had participated for three months or less (i.e. those in their first term) were excluded as they were still likely adjusting to the programme. | Psychological wellbeing | The logistic regression analysis suggested a positive association between the duration of learning and general psychological wellbeing. |
| Park et al. (2016) | Quantitative | Survey | LLL was defined as enrolment in 'Program 60', a university-based educational program for older adults over 60 years living in Ohio. Program 60 was launched in January 1974 and offers a variety of courses. Previous education background nor income does not matter for enrolment. Older students can take an unlimited number of tuition-free, non-credit, non-degree classes (including online classes) offered at Ohio State University (alongside younger students) and can also utilise other college resources. The survey included a question regarding the number of years that older learners had been involved in the program (numerical variable) and the number of classes taken (numerical variable). | General wellbeing/Quality of life | The participants reported that classes helped increase their emotional satisfaction and that they enjoyed taking classes with younger students. Their experiences in the program reliably predicted psychological and social elements of their quality of life (p=004 and p=019, respectively). |
| Pikhart & Klimova (2020) | Quantitative | Survey | LLL was defined as participation in foreign languages (English or German) courses, either at the University of Third Age or organized by the company where the participants were employees. The age-based inclusion criterion for older adults was 55 years and above. No information on participation duration was recorded. | General wellbeing/Quality of life | The results revealed that participation in learning enhanced the overall wellbeing of older people, which was shown in their expression of their feelings of happiness, satisfaction, and positive motivation to learn a foreign language. |

| | | | | | |
|---|---|---|---|---|---|
| Portero & Oliva (2007) | Quantitative | Survey | LLL was defined as individuals aged 55 and older enrolled in the Third Age University Program Aula de la Experiencia at the University of Seville during the academic year 1999–2000. No other information on LLL was recorded. | Psychological wellbeing, Physical health | The results indicated significant improvements in the participants' psychological wellbeing, perceived health, and social support networks after a year in the programme. Notably, the study established a causal link, suggesting that increased social support positively influenced psychological wellbeing, which in turn had beneficial effects on health. |
| Sabeti (2015) | Qualitative | Interviews and observations | LLL was defined as enrolment in an adult creative writing class based at a major urban art gallery in the United Kingdom. The particular class was not designed specifically for an older age group. However, because of the scheduling—ten o'clock in the morning on a Monday—it attracted a group of older adults. No specific age limits for 'later life' were defined in this study. Participants were described as 'older adults, with most of them being post-retirement and all were above the age of 55. The regular pattern of meetings was such that a tour session (in any of the three sites of the gallery), lasting approximately two hours, was followed two weeks later by a writing session of approximately two and half hours. The guides were often freelance artists, or art historians; occasionally, the group was choosing to lead the tour themselves. The writing session was always taking place at the main gallery building in the centre of the town, and it was led by a writer. The writers were employed on a freelance basis; they were published authors themselves and had experience working with creative writing groups. At the end of a tour session and halfway through a writing session, the group was having a tea break. The group had been running for many years and had remained stable throughout the time. The group met once a fortnight on a Monday morning throughout the year, other than in August when they had a summer break. | General wellbeing/Quality of life | The writer argues that the value of participating in a creative writing program in later life lies both in the fact that it is a relational (rather than individual) process and a means of being in the present. |
| Simone & Cesena (2010) | Quantitative | Survey | LLL was defined as the enrolment in adult education programs at (a) Santa Clara University's Osher Lifelong Learning Institute (OLLI) and (b) Santa Clara County Adult Education (AE). Both programs are open to any adult over the age of 50 (OLLI) or 55 (AE), and both require a fee that covers the costs of the classes. OLLI classes cost $6.00=hour and AE class costs vary but range from $2 to $10=hour. The students differed most substantially in class enrolment. The AE students participated in art and music classes, whereas the OLLI students participated in more traditional academic-topic classes such as history, literature, and biology. No information about the duration of attendance was recorded. | General wellbeing/Quality of life | Results showed that participation in non-credit lifelong learning programs boosted the mood of older adults and could offer longer-term advantages for their overall health and wellbeing. |

| | | | | | |
|---|---|---|---|---|---|
| Sloane-Seale & Kops (2008) | Qualitative | Focus group | LLL was defined as enrolment in lifelong learning programs organized by older adults' organizations. More specifically, a purposeful sampling technique was employed to recruit older learners from older adults' organizations that provided continuing education. The age-based inclusion criterion for older learners was not recorded. Participants were described as 'older adult learners', with ages ranging from 65 years and above. No information on the duration or frequency of participation was recorded. | General wellbeing/Quality of life | The study suggests that lifelong learning contributes significantly to an improved quality of life and successful aging among older adults. |
| Southcott & Li (2018) | Qualitative | Interviews and observations | LLL was defined as individuals aged 50 and older enrolled in weekly singing lessons at the Tangshan Older People University (TOPU), China. Tangshan Older People University was established in 1991 to support retired people and provides a wide range of lifelong learning opportunities in its continuing education system for people 50+ years. In 1999, weekly singing courses were formed at TOPU, and they are one of the most significant activities at TOPU. Both Western and Chinese music are being taught. In this study, most of the participants had attended for about three years, but one stayed for 20. | Psychological wellbeing, Physical wellbeing, Cognitive wellbeing | This research utilized a case study design and data were analyzed using interpretative phenomenological analysis which explores experience as understood by participants. Three broad health-related themes were identified from the data: emotional wellbeing, physical wellbeing, and mental wellbeing. |
| Wenzel et al. (2024) | Quantitative | Survey | LLL was defined as enrolment in nine Osher Lifelong Learning Institutes (OLLI) in the U.S. The primary mission of Osher-funded institutes is to provide intellectually stimulating learning opportunities for older adults. There are currently 125 OLLIs in the U.S. operating on college and university campuses in all 50 states and the District of Columbia, providing courses and activities to more than 117,000 members.<br>In general, OLLI participants are at least 50 years old and retired from employment. Individual eligibility criteria for this study included OLLI membership and being 65 years of age or older. No information about the duration of attendance was recorded. | Physical health, Cognitive health, Mental health | The study examined demographics, physical and mental health, cognitive ability, meaning in life, cognitive reserve, and successful ageing among older learners, comparing their profiles to the general older adult population in the US. The findings support the idea that participation in learning is linked to higher levels of physical and mental health, increased cognitive reserve, and a greater sense of successful aging. |
| Zadworna (2020) | Quantitative | Survey | LLL was defined as enrolment in the U3A, which aims to organize and conduct lectures, seminars, and regular classes, including workshops, courses, and interest circles, and to provide cultural and artistic input. Its objective is to enhance the quality of life through educational, social, and sporting activities. No information about the activities or the duration of attendance was recorded. The age-based inclusion criterion in the study was a minimum age of 60 years. | Physical health, Health-promoting behaviors | The U3A attendees presented significantly higher scores for general health behavior and some of its components, and declared higher self-rated health than their peers not affiliated with any educational organization. |

**Observational - investigation of more general engagement in learning activities**

| Reference | Design | | Operationalization and/or measurement of LLL | Health-related outcomes | Key findings |
|---|---|---|---|---|---|
| Jenkins & Mostafa (2015) | Quantitative | Longitudinal cohort survey | LLL was defined as self-reported engagement in formal and informal learning activities. Specifically, the study used data from the English Longitudinal Study of Aging, a continuing, longitudinal survey of adults | General wellbeing/Quality of life | Learning was associated with higher wellbeing after controlling for a range of factors. The results showed that |

| | | | | | |
|---|---|---|---|---|---|
| | | | aged 50 and above. Respondents self-reported participation (dichotomous variable) in one or more of four different types of learning activities every two years: 1) Formal learning that leads to a qualification, 2) Formal education/training courses, 3) Informal learning such as education, music, arts groups or evening classes, and 4) Informal learning such as sports club, gym or exercise classes. For the study, researchers selected individuals who were aged 50 to 69 at Wave 1. They used measures of engagement in LLL for up to 7 years. | | informal types of learning were associated with higher wellbeing. There was no evidence that formal education/training courses were associated with higher wellbeing. |
| Sloane-Seale & Kops (2010) | Quantitative | Survey | LLL was defined as self-reported engagement in formal and informal learning.<br>The ‘Older Adults in Lifelong Learning & Successful Aging’ survey was administered to a stratified random sample of older adults aged 55 and older, which was drawn from the database of Manitoba Health. Respondents self-reported participation (dichotomous variable) in both formal and informal educational activities in the past two years since the data collection.<br>For the purposes of the article, formal education was defined as education that is normally organized by educational and non-educational institutions in the form of short-term/ non-credentialed courses, workshops, seminars, and lectures. Informal education was defined as education that is normally self-planned, whereby individuals or groups learn in a variety of ways using a variety of resources. Respondents were also asked to report their subject interests (multiple-choice list). | Physical health, Psychological wellbeing | The results suggest that participation in learning activities leads to a more inclusive and comprehensive understanding of successful aging; that educational activities positively influence mental and physical activity, which in turn result in more positive health and wellbeing; and that spirituality and life planning, including a positive sense of self, a focus on personal renewal and growth, a connection to the broader community, and setting life goals, contribute to successful aging. |
| Tam & Chui (2016) | Quantitative | Survey | LLL was defined as self-reported engagement in organized formal and non-formal learning.<br>Older adults recruited from a network of 17 community centres spread across Hong Kong and administered the ‘Learning and Ageing Survey 2013’. Respondents self-reported participation (dichotomous variable) in both formal and non-formal learning that was organized by educational and non-educational institutions or organizations in the form of courses, workshops, seminars, and lectures, for up to 6 months since the data collection. They were also asked to report their subject interests (multiple-choice list). No specific age limits for ‘later life’ were reported. Participants were described as ‘elders’, with ages ranging from 55 years and above. | General wellbeing/Quality of life | The findings showed that older learners strongly agreed that "Learning keeps me healthy physically and mentally". This was one of the top three most strongly agreed-upon meanings of later life learning, indicating a direct perceived health benefit of engaging in learning activities. The study also found a significant positive correlation between participation in learning and self-reported general health condition. Between 'learners' and 'non-learners', 'learners' more strongly perceived that learning contributes to their physical and mental wellbeing. |
| Wang et al. (2018) | Mixed | Survey | LLL was defined as self-reported engagement in formal, non-formal, and informal learning activities.<br>Older adults recruited from U3As, older adults’ centres, and residential communities, and were administered a survey. Respondents self-reported participation (5-point Likert scale ranging from 1 [never], 2 [once | Physical wellbeing, Psychological wellbeing, Social | The results indicated that being engaged in learning activities in later life has the potential to increase the quality of life of older adults. Moreover, in contrast to nonformal learning, the |

| | | | | | |
|---|---|---|---|---|---|
| | | | half a year], 3 [monthly], 4 [weekly], and 5 [daily]) in one or more of three different types of learning:<br>(1) formal learning (i.e., participation in the U3As and participation in online educational programs);<br>(2) nonformal learning (i.e., participation in community-organized educational programs, participation in club educational programs, and participation in broadcasting educational programs); and<br>(3) informal learning activities (i.e., unplanned, everyday occurrences, e.g., reading books/magazines, watching TV programs, surfing the Internet, seeking advice from peers/family members, participation in communities leisure/volunteer activities, spontaneous sports or leisure exercise, going to library or museum, travelling, including outbound travel, domestic travel, and outing).<br>No specific age limits for 'later life' were reported. Participants were described as 'older adults', with ages ranging from 52 years and above. | wellbeing, Economic wellbeing | results of the hierarchical linear regression model demonstrated that both informal and formal learning had a significant positive influence on quality of life. |
| **Interventional - investigation of a pre-existing, specified learning activity** | | | | | |
| **Reference** | **Design** | | **Operationalization and/or measurement of LLL** | **Health-related outcomes** | **Key findings** |
| Bužgová et al. (2024) | Quantitative | Quasi-experimental design - one-group intervention | LLL was defined as adults aged 60 and over participating in the 'Living More Healthily and Actively' program at the U3A, University of Ostrava, Czech Republic, for one academic year.<br>The program aims to increase the knowledge of older people in the field of health, healthy lifestyle and disease prevention, motivating them to lead a healthy and active life, thereby contributing to increasing the quality of life in later life and promoting prosperity in an ageing society.<br>The annual cycles included lectures on: 1.Cardiovascular Diseases, 2.Mental Health, and 3.Neurological Diseases.<br>In total, older people attended 16 lectures and two practical lessons in each educational module. The lectures were run once every two weeks for 90 minutes. Practical lessons were included after the whole series of lectures and lasted 60 minutes. The lectures and practical lessons were led by experienced academic staff from the Faculty of Medicine of University of Ostrava. The attendance of the participants in the training program was monitored. | Mental health | While the course did not show statistically significant improvements across the entire participant group, the study identified meaningful positive effects for individuals who initially presented with poorer scores in areas like depression and anxiety. |
| Dias et al. (2017) | Quantitative | Quasi-experimental design | LLL was defined as participation in a health education program in a University of Third Age in Brazil.<br>For this study, the inclusion criterion based on age was set at 50 years or older. The education program consisted of 10 sessions with group educational and dynamic actions, including orientations on disease prevention and cognitive stimulation exercises. The themes of the program focused on gerontology, the cardiac system, mental health, | Cognitive health, Physical health, Mental health | Significant improvements were observed for the learning group (intervention group) when comparing the total ACE-R score (p=0.001) and memory domain (p=0.011) before and after the learning intervention. |

| | | | | | |
|---|---|---|---|---|---|
| | | | cognitive ageing, musculoskeletal ageing, integumentary ageing, supplementation and vitamins, and physical activity.<br>The program lasted 4 months. Sessions were held once every week and were lasting for an hour. Attendance was monitored. | | |
| Fernández - Ballesteros et al. (2012) | Quantitative | Quasi-experimental design | LLL was defined as enrolment in the University Program for Older Adults (PUMA) at the Autonomous University of Madrid.<br>The goals of the PUMA program are: (1) to promote knowledge and competencies (measured by tests and exams), (2) to promote personal development, and (3) to increase social participation. To access PUMA, applicants have to be aged 55 or older and pass an academic exam. The PUMA program is of three academic years' duration with a total of 450 teaching hours. The program offers a variety of courses. Attendance at lectures is mandatory. At the end of each course, students have an achievement evaluation.<br>In the particular study, the experimental group consisted of older students, aged between 55 and 70 years, who were enrolled in PUMA from 2007 to 2011. Attendance was monitored. | Physical health, Cognitive health, Mental health, Social health | Pre/post comparisons showed that participants obtained significant benefits, attributable to the program, in that they maintained their cognitive performance evaluated through the Digit-Symbol Test, their health (assessed through the number of illnesses reported), and their level of activity (information-seeking and social activities), and increase their level of positive affect. At the end of the program, significantly more of those who enrolled on it were classified as "active agers," compared to the control group. |
| Lenehan et al. (2016) | Quantitative | Quasi-experimental design | LLL was defined as engagement in higher education (i.e., university undergraduate courses) for a minimum period of 12 months. No specific age limits for 'later life' were defined. Participants were described as 'older adults' with ages ranging from 50 to 79 years of age at the commencement of the study. Information about the courses was not recorded. Attendance was monitored. | Cognitive health | Results demonstrated that 44.3% of the control group showed no change in cognitive reserve, whereas 92.5% of the further education participants (intervention group) displayed a significant linear increase in cognitive reserve over the 4 years of the study. |
| Panayotoff (1993) | Quantitative | Quasi-experimental design | LLL was defined as participation in one of four different continuing educational programs for older adults at the Northampton County Community College in Bethlehem, Pennsylvania:<br>1) the Late Start program that was restricted to first-time enrolees as an orientation to the college), 2) the Educational Growth Opportunities program that emphasizes academics in a small-group approach, 3) Update, the largest program, that had a practical and academic focus, and 4) the Senior Camaraderie program that dealt more with older adult concerns, hobbies, and travel.<br>All programs were being offered in the fall and spring of each year. No specific age limits for 'later life' were defined. Participants were described as 'older adults', with mean ages in each program ranging from 66 to 69. No information about the duration of attendance was recorded. Participation was defined based on recruitment of older adults enrolled in one of the four programs. | Mental health | The findings indicated short-term changes in depression, social satisfaction, and symptoms of aging, with some programs showing improvement and others decline. However, these effects did not persist six weeks after the programs concluded, suggesting the benefits are not long-lasting. |

| | | | | | |
|---|---|---|---|---|---|
| Richeson et al. (2007) | Mixed | Quasi-experimental design - one-group intervention | LLL was defined as the participation in the 'Health Promotion for the Mind, Body, and Spirit' program, an adult education course designed for people with a diagnosis of mild cognitive disorder or early-stage dementia.<br>The age-based inclusion criterion was older adults who were 60 years and older.<br>The program was part of the Osher Lifelong Learning Institute (OLLI) at the University of Southern Maine, Portland, USA. It was designed to provide information on the disease process and on healthy behaviours to prevent problems that are common later in the course of the disease. It included modules on physical and cognitive fitness, nutrition, recreation, communication, understanding the disease process, depression, coping, relationships, and home and travelling safety. The cost was free to the participants.<br>The course lasted 13 weeks, and it was held on Fridays from 9:30 to 11:30 a.m. Attendance was monitored. | Cognitive health, Mental health | Key findings revealed a significant improvement in participants' general perceived self-efficacy scores. While no significant differences were found in MMSE or GDS15 scores, a slight increase in MMSE mean scores and a slight decrease in depression mean scores were observed. |

**Interventional - investigation of a learning intervention designed by the researchers**

| Reference | Design | | Operationalization and/or measurement of LLL | Health-related outcomes | Key findings |
|---|---|---|---|---|---|
| Bubbico et al. (2019) | Quantitative | Randomized control design | LLL was defined as participation in a foreign language (English) learning program designed for older adults.<br>No specific age limits for 'later life' were defined. Participants were described as 'elderly', with ages between 59 and 79 years. The aim of the program was for students to learn basic vocabulary and grammar in areas such as traveling, shopping, and family. British and American English traditions, customs, and culture were also taught. Students had to work on team projects in order to improve their oral and written communication in English.<br>The program lasted 4 months and comprised 16 weekly sessions. Each training week consisted of 1 hour and a half classroom session, interspersed with a 15-minute break and half an hour of homework exercises. All participants were assessed qualitatively by their native teacher at the beginning and at the end of the course. Attendance was monitored. | Cognitive health | At the end of the program, in the learning group (intervention group) the results showed a significant improvement in global cognition together with an increased functional connectivity in the right inferior frontal gyrus, right superior frontal gyrus and left superior parietal lobule. |
| Cusack et al. (2003) | Quantitative | Quasi-experimental design - one-group intervention | LLL was defined as participation in the Mental Fitness for Life program, an 8-week program of intensive workshops that includes the following topics: Goal Setting; Critical Thinking; Creativity; Positive Mental Attitude; Learning; Memory; and Speaking your Mind.<br>The program is offered to people aged 50 who are concerned about memory and want to improve their mental abilities. Participants learn how ageist attitudes and beliefs about declining mental abilities restrict their potential for a vital, healthy later life. They also learn how to set | Cognitive health, Mental health | The Mental Fitness for Life program significantly impacted mental health by reducing depression and enhancing various aspects of mental fitness, contributing to overall wellbeing and a vital, healthy aging experience. |

| | | | | | |
|---|---|---|---|---|---|
| | | | meaningful goals and achieve them, how to change negative beliefs to positive ones, to think critically and creatively, to appreciate diversity and different perspectives, and to take risks that extend and enrich their lives. The sessions are stimulating and challenging with quizzes, puzzles, assignments, and provocative dialogue and debate. Attendance was monitored. | | |
| Díaz-López et al. (2016) | Quantitative | Randomized control design | LLL was defined as participation in a new information and communication technology education program for individuals over the age of 55. Specifically, the study was focused on a specific group of older adults who were engaged in Active Participation Centres that offer leisure time activities for older people in Almería, Andalusia, Spain. The program lasted over four months and was carried out in two weekly sessions of an hour and a half each. An initial block of activities focused on content that was related to mobile telephones. The second block consisted of the use of credit cards, bank operations and purchases over the Internet. The final block was dedicated to computer-related activities. Activities were carried out in small groups, pairs, and individuals. Attendance was monitored. | General wellbeing/Quality of life | Results revealed a high degree of participant satisfaction (76.6 %), as well as improvements in quality of life as compared to the control group after the 3-month program. |
| Escolar Chua & De Guzman (2014) | Quantitative | Randomized control design | LLL was defined as participation in community-based educational programs designed for older adults aged between 60 and 80 years. The programs were designed based on a needs assessment interview conducted in the community before the start of the study and comprised of a mix of activities that catered to the needs of the Filipino older adults for physical activity, mental stimulation, social engagement, health promotion and development of new skills. Three main programs were implemented: (a) a wellness program (b) a physical fitness activity program, and (c) livelihood training program. The programs were planned, developed, and facilitated by volunteer professors from a local university and were offered for four months. Attendance was monitored. | Mental health | The results of t tests showed statistically significant group differences between the intervention and control group, with the learning group (intervention) showing higher life satisfaction, self-esteem, and lower depression levels compared to the control group. |
| Fitzsimmons & Buettner (2003) | Mixed | Quasi-experimental design - one-group intervention | LLL was defined as participation in an experimental college course, “Health Promotion for the Mind, Body, and Spirit”, for older adults 65 years of age or older with newly diagnosed dementia. The course was designed to provide information on the disease process and on healthy behaviours to prevent problems that are common later in the course of the disease. It was designed in 10 weekly modules (class was held on Wednesdays from 10:00 a.m. to noon), with different experts and students providing information each week. Attendance was monitored. Course topics covered over the 10-week period, included modules on: healthy lifestyles, dementia, depression, delirium, cognitive activities, communication relationships and coping, physical fitness, nutrition & | Mental health, Health-promoting behaviors | The course showed a promising impact on participants' mental health. Participation in the course led to reported short-term changes in lifestyle and health behaviours among individuals with early-stage dementia. The course fostered a strong sense of camaraderie among participants, who bonded, helped, consoled, complimented, and hugged each other. |

| | | | | | |
|---|---|---|---|---|---|
| | | | hydration, medications recreation & leisure, home and traveling safety, lifelong learning, and future planning and graduation. | | |
| Fu et al. (2018) | Quantitative | Quasi-experimental design - one-group intervention | LLL was defined as participation in a group-singing program in the community, which was developed by the research team.<br>The age-based inclusion criterion was set to 60 years and above.<br>Participants' song preferences were assessed at study entry and were added to the customized song repertoire for each group.<br>The program lasted for three months and consisted of 12 weekly 75-min sessions. Each session consisted of a 10-min pre-singing activity (muscle stretching, deep breathing, and vocal exercise) and 50-min song-singing/ learning, followed by 15-min informal social time and refreshments. Attendance was monitored. | Cognitive health, Physical health, General wellbeing/Quality of life | Results revealed a significant improvement in phonological ($p < 0.0001$) and animal ($p=0.0004$) semantic Verbal Fluency Tests, immediate Word Recall Test ($p < 0.0001$), Maximum Inspiratory Pressure ($p=0.0001$), Maximum Expiratory Pressure ($p < 0.0001$), and in-session oxygen saturation ($p=0.03$). |
| Hsu et al. (2023) | Mixed | Quasi-experimental design - one-group intervention | LLL was defined as participation in a learning intervention.<br>The researchers developed a game-based health promotion program, KABAN!, for older adults with integrated health domains (improving health literacy, quality of life, and positive emotions) through gamification design and experiential learning concepts. No specific age limits for 'later life' were defined. Participants were described as 'older adults', with ages between 55 and 106 years. No information was recorded on duration and frequency.<br>The motivational factors in KABAN! were composed of storylines and achievements. The main storyline was adapted to one of the most popular activities among Taiwanese older adults, mountain hiking. KABAN! consisted of six stages of challenges created for twenty small activities within 120 minutes long. Instructors and participants set up the venue layout in various settings, such as the local community centers, in front of a temple or in any open space areas. They also dressed up like hiking guides with flags, a big mountain trail map, and banners. Each instructor carried one suitcase per session.<br>Each session consisted of 20 to 30 participants with all instructional materials and props packed in a portable suitcase, making it easier to carry around in different communities, including the city and countryside. An immersive experience was implemented throughout the program, including physical exercises, cooking games for nutritional knowledge, social interaction, cognitive training, and oral health care skills. | Psychological wellbeing | Analyses revealed that health literacy levels, positive emotions, and quality of life significantly increased after participation, while negative emotions remained identical. Further investigation of qualitative data via the constant comparison method showed positive feedback on instructors' perceived changes in participants. |
| Johnson (2014) | Mixed | Quasi-experimental design - one-group intervention | LLL was defined as participation in an intergenerational intervention in which older adults, who were enrolled in an Adult Day program located in the Republic of Trinidad and Tobago, learned specified digital communication technologies from older adolescents (18 – 21 years old). The age-based inclusion criterion for older adults was between 65 and 80 years. Each adolescent was paired with an older adult and guided them | Psychological wellbeing | The study employed a mixed-methods approach, combining quantitative data from scales measuring depression, life satisfaction, and quality of life, with qualitative insights from open-ended surveys and focus groups. Quantitative |

| | | | | | |
|---|---|---|---|---|---|
| | | | in learning the following technologies: logging onto the internet, setting up email accounts, using Facebook, Instant Messaging, and conducting Internet searches.<br>The intervention lasted for 6 weeks and consisted of six 2-hour sessions. Attendance was monitored. | | results showed no significant differences; the qualitative findings illuminated the experiences and perceptions of ageing among the participants, particularly regarding social connectedness, health concerns, financial considerations, and cultural values. |
| Kao & Chang (2017) | Quantitative | Randomized control design | LLL was defined as participation in a leisure educational program designed to improve the leisure attitudes, leisure knowledge, and leisure skills of participants, thereby allowing them to use their leisure time more effectively and satisfactorily.<br>The age-based inclusion criterion for older adults was 65 years and above. The educational program consisted of 12 units, each of which comprised a variety of activities such as discussion exercises, role-playing, and participation in leisure activities. All units and demonstrated activities were delivered at the Department of Living Sciences, National Open University, New Taipei, Taiwan. The training was conducted twice per week for 3 months. The participants spent approximately 2 hours participating in each unit. Attendance was monitored. | Mental health | The study found that the education program significantly reduced stress in older adults. The average post-test and follow-up test scores of stress in the experimental group were significantly lower than both their pre-test scores and the corresponding scores in the control group. These lower levels of stress were sustained a year later, indicating a long-term benefit. |
| MacRitchie et al. (2020) | Mixed | Randomized control design | LLL was defined as participation in a piano training program that was designed by the research team.<br>The inclusion criteria for older adults were that they had to be aged 65 years or over, with less than 2 years of formal music instrument training. The training program lasted 10 weeks and consisted of 10 lessons, each of 60 minutes duration, supplemented by at-home practice specified at 30 min per day (total intervention duration was approximately 600 + 1800 min). Attendance was monitored.<br>All lessons incorporated three main elements to a varying degree: (i) exercises/warm-ups, (ii) playing of melodies, and (iii) ensemble playing tasks. | Cognitive health, Physical health | Key findings demonstrated moderate evidence of a strong positive impact of the training on visuo-motor skills. However, moderate evidence for negative impacts was found suggesting no benefit for cognitive switching. The training showed no evidence of significant positive or negative impact on other fine motor skills or visuomotor coordination. Qualitative results revealed that the group learning environment motivated participants to play in musical ensembles and to socialize. Participants experienced feelings of achievement, competence, and increased self-efficacy after the program. |
| Miller et al. (2002) | Quantitative | Randomized control design | LLL was defined as participation in a nutrition education intervention, which aimed at teaching participants how to evaluate the nutrition information on food labels for food purchasing, meal planning, and diabetes management.<br>The age-based inclusion criterion for older adults was 65 years and above. The intervention included 10 weekly group sessions. Each | Physical health | Participants exceeded the guidelines for optimal glycaemic control at pretest. The learning group (intervention group) had greater improvements in fasting plasma glucose ($P = 0.05$) and glycated haemoglobin ($P < 0.01$) than the control |

| | | | | | |
|---|---|---|---|---|---|
| | | | session lasted 1.5 to 2 hours and was led by the same registered dietitian. At each group session, participants set a weekly goal and reward to facilitate and reinforce behaviour change. Attendance was monitored. | | group. Significantly more participants in the intervention group than control group met the treatment goals for total cholesterol at post-test (P < 0.05). |
| Perkins & Williamon (2014) | Mixed | Quasi-experimental design | LLL was defined as participation in a music learning intervention conducted as part of the Rhythm for Life project run by the authors at the Royal College of Music, London, UK.<br>The intervention lasted for 10 weeks, with weekly lessons lasting 1 to 2 hours each, and was offered free of charge to adults aged 50 years and above. Each participant joined one of three different programmes designed for musical beginners:<br>Program 1: a 10-week program of free one-to-one instrumental lessons on keyboard, guitar, recorder or djembe drum.<br>Program 2: a 10-week program of small-group instrumental lessons on keyboard, guitar, recorder or djembe drum. Groups ranged from three to eight participants. In both programs, instruments were provided for each learner which they kept following the conclusion of the program.<br>Program 3: a 10-week program of creative music workshops, led by an RCM alumnus for approximately 20 learners, with support from four RCM students.<br>Attendance was monitored. | General wellbeing/Quality of life, Health-promoting behaviors | Findings revealed general improvements in wellbeing across all learning groups, with music learners of higher socioeconomic status showing a steeper increase in physical activity and spiritual growth. |
| Santini et al. (2020) | Qualitative | Quasi-experimental design - one-group intervention | LLL was defined as participation in the 'Green Care Program', an informal educational program delivered by six farms in central Italy for the enhancement of physical health, psychological wellbeing, social engagement, and lifelong learning among frail older adults.<br>The age-based inclusion criterion for older adults was 65 years and older.<br>An informal educational methodology was adopted by skilled psychologists, based on learning by doing, self-narration, and experiential learning.<br>The types of educational activities were chosen according to the farms' productive characteristics (e.g., if the farm had an orchard, participants could prune trees, and if the farm cultivated medicinal herbs, they learned how to make essential oils), and to the participants' expectations and physical condition (i.e., lighter activities for daycare centre users and a slightly more intense activities for people living in the community).<br>Activities were delivered in group sessions, and each group had one or two tutors. The program was delivered over 50 full days (about 6 hours a day) of training on the farms for 12 months. Attendance was monitored. | Physical health, Psychological wellbeing, Social health | Individuals' perception of the training benefits varied depending on their health and social condition at baseline. The program enhanced the perceived wellbeing and health of day care centre users. |
| Seinfeld et al. (2013) | Quantitative | Quasi-experimental design | LLL was defined as participation in a piano training intervention.<br>The age-based inclusion criterion was 60 years or older. More specifically, group piano lessons, lasting one hour and a half, were given in a community centre on a weekly basis by the same music teacher who had designed the program. Attendance was monitored. | Cognitive health, Psychological wellbeing | The findings suggested that piano training significantly improved executive function and showed positive trends in visual scanning and motor ability. Additionally, the study indicated that |

| | | | | | |
|---|---|---|---|---|---|
| | | | The classes combined essential theoretical knowledge about music notation and theory with actual practice of piano playing. Participants were committed to practice independently at least 45 min per day at least 5 days per week (~4 h per week). Each day they had to practice playing a piano sequence 10 times with their dominant hand, and 10 more times with their non-dominant hand. Subjects had free access to practice the piano during all the week in the community centre. The level of difficulty of the piano program increased gradually. | | piano lessons led to decreased depression and enhanced psychological wellbeing. |
| Shapira et al. (2007) | Mixed | Quasi-experimental design | LLL was defined as participation in a tailored Computer operation and Internet use educational program that aimed at equipping older adults with skills for operating a personal computer and using several Internet applications, including handling e-mail, browsing the Web and exploiting different types of sites and participating in forums and virtual communities. No specific age limits for 'later life' were defined. Participants were described as 'older people', with ages ranging from 55 years and above. The program lasted 15 weeks and included one or two lessons per week, each approximately 60 minutes long. The instructors, veteran teachers in the use of computers and Internet, were especially experienced in working with older people. They were assisted by volunteers, who provided participants with additional help and guidance when needed. Attendance was monitored. | Psychological wellbeing | The finding revealed that learning and using the Internet significantly improved older adults' life satisfaction, sense of control, and reduced feelings of depression and loneliness. The study highlights that these benefits extend beyond preventing age-related decline, actively enhancing psychological factors crucial for quality of life. The research also explored the qualitative aspects of this improvement, noting participants' pride in learning new technology, enhanced social connections, and increased feelings of involvement and positive emotions. |
| Shokouhi et al. (2019) | Quantitative | Randomized control design | LLL was defined participation in an educational intervention on oral health related quality of life of older adults. No specific age limits for 'later life' were defined. Participants were described as 'elderly' over 60 years. The training program comprised of a combination of in-person training (individual training through motivational interviewing and group discussion) and non-attendance training (one week following the face-to-face training, educational messages were sent once a week) and lasted for one month. Attendance was monitored. | Physical health | Educational intervention was significant in terms of overall oral health related quality of life and the overall effectiveness score of adult learning theory ($P < 0.001$). There was a significant difference between the two groups in terms of the mean change score of three physical, psychosocial, and pain dimensions following the educational intervention ($P < 0.001$). |
| Uemura et al. (2021) | Quantitative | Randomized control design | LLL was defined as participation in an active learning education program on arterial stiffness. The learning program was designed by the research team with the aim to promote health literacy and physical activity. No specific age criterion was defined. Participants were described as 'older adults', with ages ranging from 65 years and above. The program involved learning in higher-order thinking (e.g., evaluation, synthesis) and participation in activities (e.g., discussion, presentation, planning) rather than passive listening. The themes of the program focused on the role of exercise, diet/nutrition, and cognitive activity in | Physical health, Health-promoting behaviors | The analysis of data revealed that the learning group (intervention group) showed significant improvement in arterial stiffness (between-groups difference = -0.78, Cohen's d=0.82) and physical activity (between-groups difference = 32.5 MET-h/week, Cohen's d=0.57). |

| | | | | | |
|---|---|---|---|---|---|
| | | | health promotion among older adults. This program aimed to promote behaviour changes in daily life and self-management in accordance with an individual's health status. The program lasted 24 weeks and consisted of weekly 90-min sessions. Attendance was monitored. | | |
| Valis et al. (2019) | Quantitative | Randomized control design | LLL was defined as participation in an English language training intervention, which should lead to the improvement of cognitive functions among healthy older individuals. The age-based inclusion criterion was 55 years and above. The intervention period lasted for 12 weeks, and the participants altogether had 36 English lessons. Attendance was monitored. The participants in the experimental group had three 45-min lessons once a week, every Wednesday. The program was aimed at developing all four language skills, and special attention was given to developing and practising vocabulary and phrases under the supervision of two experienced English language teachers. Traditional (e.g., drilling or repetition of vocabulary) and non-traditional (e.g., mind mapping or problem-solving) methods of teaching were used. | Cognitive health | The results of the research showed that there had been a slight enhancement of cognitive skills in the learning group (intervention group). Nevertheless, overall, the scores of the experimental and control groups did not considerably differ. |

*Note*. LLL = Later life learning; U3A = University of the Third Age.

**Preferred Reporting Items for Systematic reviews and Meta-Analyses extension for Scoping Reviews (PRISMA-ScR) Checklist**

| SECTION | ITEM | PRISMA-ScR CHECKLIST ITEM | REPORTED ON PAGE # |
|---|---|---|---|
| **TITLE** | | | |
| Title | 1 | Identify the report as a scoping review. | 1 |
| **ABSTRACT** | | | |
| Structured summary | 2 | Provide a structured summary that includes (as applicable): background, objectives, eligibility criteria, sources of evidence, charting methods, results, and conclusions that relate to the review questions and objectives. | 2 |
| **INTRODUCTION** | | | |
| Rationale | 3 | Describe the rationale for the review in the context of what is already known. Explain why the review questions/objectives lend themselves to a scoping review approach. | 3-4 |
| Objectives | 4 | Provide an explicit statement of the questions and objectives being addressed with reference to their key elements (e.g., population or participants, concepts, and context) or other relevant key elements used to conceptualize the review questions and/or objectives. | 4 |
| **METHODS** | | | |
| Protocol and registration | 5 | Indicate whether a review protocol exists; state if and where it can be accessed (e.g., a Web address); and if available, provide registration information, including the registration number. | 5 |
| Eligibility criteria | 6 | Specify characteristics of the sources of evidence used as eligibility criteria (e.g., years considered, language, and publication status), and provide a rationale. | 5-6 |
| Information sources* | 7 | Describe all information sources in the search (e.g., databases with dates of coverage and contact with authors to identify additional sources), as well as the date the most recent search was executed. | 6 |
| Search | 8 | Present the full electronic search strategy for at least 1 database, including any limits used, such that it could be repeated. | 45-47 |
| Selection of sources of evidence† | 9 | State the process for selecting sources of evidence (i.e., screening and eligibility) included in the scoping review. | 6 |
| Data charting process‡ | 10 | Describe the methods of charting data from the included sources of evidence (e.g., calibrated forms or forms that have been tested by the team before their use, and whether data charting was done independently or in duplicate) and any processes for obtaining and confirming data from investigators. | 7 |

| SECTION | ITEM | PRISMA-ScR CHECKLIST ITEM | REPORTED ON PAGE # |
|---|---|---|---|
| Data items | 11 | List and define all variables for which data were sought and any assumptions and simplifications made. | 6-7 |
| Critical appraisal of individual sources of evidence§ | 12 | If done, provide a rationale for conducting a critical appraisal of included sources of evidence; describe the methods used and how this information was used in any data synthesis (if appropriate). | n/a |
| Synthesis of results | 13 | Describe the methods of handling and summarizing the data that were charted. | 7-8 |
| **RESULTS** | | | |
| Selection of sources of evidence | 14 | Give numbers of sources of evidence screened, assessed for eligibility, and included in the review, with reasons for exclusions at each stage, ideally using a flow diagram. | 8-9, 43 |
| Characteristics of sources of evidence | 15 | For each source of evidence, present characteristics for which data were charted and provide the citations. | 49-53, 23 |
| Critical appraisal within sources of evidence | 16 | If done, present data on critical appraisal of included sources of evidence (see item 12). | n/a |
| Results of individual sources of evidence | 17 | For each included source of evidence, present the relevant data that were charted that relate to the review questions and objectives. | 49-53, 54-70 |
| Synthesis of results | 18 | Summarize and/or present the charting results as they relate to the review questions and objectives. | 9-15, 33-42 |
| **DISCUSSION** | | | |
| Summary of evidence | 19 | Summarize the main results (including an overview of concepts, themes, and types of evidence available), link to the review questions and objectives, and consider the relevance to key groups. | 16-21 |
| Limitations | 20 | Discuss the limitations of the scoping review process. | 21 |
| Conclusions | 21 | Provide a general interpretation of the results with respect to the review questions and objectives, as well as potential implications and/or next steps. | 21 |
| **FUNDING** | | | |
| Funding | 22 | Describe sources of funding for the included sources of evidence, as well as sources of funding for the scoping review. Describe the role of the funders of the scoping review. | 22 |

JBI = Joanna Briggs Institute; PRISMA-ScR = Preferred Reporting Items for Systematic reviews and Meta-Analyses extension for Scoping Reviews.

* Where *sources of evidence* (see second footnote) are compiled from, such as bibliographic databases, social media platforms, and Web sites.

† A more inclusive/heterogeneous term used to account for the different types of evidence or data sources (e.g., quantitative and/or qualitative research, expert opinion, and policy documents) that may be eligible in a scoping review as opposed to only studies. This is not to be confused with *information sources* (see first footnote).

‡ The frameworks by Arksey and O'Malley (6) and Levac and colleagues (7) and the JBI guidance (4, 5) refer to the process of data extraction in a scoping review as data charting.

§ The process of systematically examining research evidence to assess its validity, results, and relevance before using it to inform a decision. This term is used for items 12 and 19 instead of "risk of bias" (which is more

applicable to systematic reviews of interventions) to include and acknowledge the various sources of evidence that may be used in a scoping review (e.g., quantitative and/or qualitative research, expert opinion, and policy document).

*From:* Tricco AC, Lillie E, Zarin W, O'Brien KK, Colquhoun H, Levac D, et al. PRISMA Extension for Scoping Reviews (PRISMAScR): Checklist and Explanation. Ann Intern Med. 2018;169:467–473. doi: 10.7326/M18-0850.
